# Simultaneous loading of PCR-based multiple fragments on mouse artificial chromosome vectors in DT40 cell for gene delivery

**DOI:** 10.1038/s41598-022-25959-9

**Published:** 2022-12-16

**Authors:** Kyotaro Yamazaki, Kyosuke Matsuo, Akane Okada, Narumi Uno, Teruhiko Suzuki, Satoshi Abe, Shusei Hamamichi, Nanami Kishima, Shota Togai, Kazuma Tomizuka, Yasuhiro Kazuki

**Affiliations:** 1 Department of Chromosome Biomedical Engineering, Integrated Medical Sciences, Graduate School of Medical Sciences, Tottori University, 86 Nishi-cho, Yonago, Tottori 683-8503 Japan; 2 Department of Biomedical Science, Institute of Regenerative Medicine and Biofunction, Graduate School of Medical Sciences, Tottori University, 86 Nishi-cho, Yonago, Tottori 683-8503 Japan; 3 Chromosome Engineering Research Center, Tottori University, 86 Nishi-cho, Yonago, Tottori 683-8503 Japan; 4 Laboratory of Bioengineering, Faculty of Life Sciences, Tokyo University of Pharmacy and Life Sciences, 1432-1 Horinouchi, Hachioji, Tokyo 192-0392 Japan; 5 Stem Cell Project, Tokyo Metropolitan Institute of Medical Science, Kamikitazawa, Setagaya-ku, Tokyo, 156-8506 Japan; 6 Department of Chromosome Biomedical Engineering, Institute of Regenerative Medicine and Biofunction, Graduate School of Medical Sciences, Tottori University, 86 Nishi-cho, Yonago, Tottori 683-8503 Japan; 7 Department of Chromosome Biomedical Engineering, School of Life Science, Faculty of Medicine, Tottori University, 86 Nishi-cho, Yonago, Tottori 683-8503 Japan; 8 Chromosome Engineering Research Group, The Exploratory Research Center on Life and Living Systems (ExCELLS), National Institutes of Natural Sciences, 5-1 Higashiyama, Myodaiji, Okazaki, Aichi 444-8787 Japan

**Keywords:** Biotechnology, Gene delivery

## Abstract

Homology-directed repair-mediated knock-in (HDR-KI) in combination with CRISPR-Cas9-mediated double strand break (DSB) leads to high frequency of site-specific HDR-KI. While this characteristic is advantageous for generating genetically modified cellular and animal models, HDR-KI efficiency in mammalian cells remains low. Since avian DT40 cells offer distinct advantage of high HDR-KI efficiency, we expanded this practicality to adapt to mammalian research through sequential insertion of target sequences into mouse/human artificial chromosome vector (MAC/HAC). Here, we developed the simultaneous insertion of multiple fragments by HDR method termed the simHDR wherein a target sequence and selection markers could be loaded onto MAC simultaneously. Additionally, preparing each HDR donor containing homology arm by PCR could bypass the cloning steps of target sequence and selection markers. To confirm the functionality of the loaded HDR donors, we constructed a MAC with human leukocyte antigen A (*HLA-A*) gene in the DT40 cells, and verified the expression of this genomic region by reverse transcription PCR (RT-PCR) and western blotting. Collectively, the simHDR offers a rapid and convenient approach to generate genetically modified models for investigating gene functions, as well as understanding disease mechanisms and therapeutic interventions.

## Introduction

Homology-directed repair (HDR) is a DNA repair mechanism that seamlessly repairs double-strand break (DSB) using homologous sequences, and HDR-mediated knock-in (HDR-KI) has been frequently used to generate genetically modified models in combination with CRISPR-Cas9^1,2^. However, while HDR-KI is frequently performed on target cells and induced pluripotent stem cells to generate cellular models^3,4^, as well as embryonic stem (ES) cells and fertilized eggs for animal models^5^, HDR-KI remains significantly inefficient with high frequency of random integration and non-homologous end joining^6,7^. Compared with the aforementioned cell lines^8,9^, avian DT40 cells have the great advantage of HDR efficiency which is higher than the mammalian cell lines. In mouse ES cells, which have the highest HDR-KI efficiency among the mammalian cells, the HDR-KI efficiency is approximately 0.5%, and increases to 4–15% when combined with CRISPR/Cas9-mediated DSB^5,10^. The HDR-KI of a gene of interest (GOI) into the DT40 cells is exceptionally efficient at approximately 50% without CRISPR/Cas9-mediated DSB^8^. The HDR-KI efficiency with CRISPR/Cas9-mediated DSB in the DT40 cells is expected to be even higher, but this hypothesis has not been tested.

**Figure 1:**
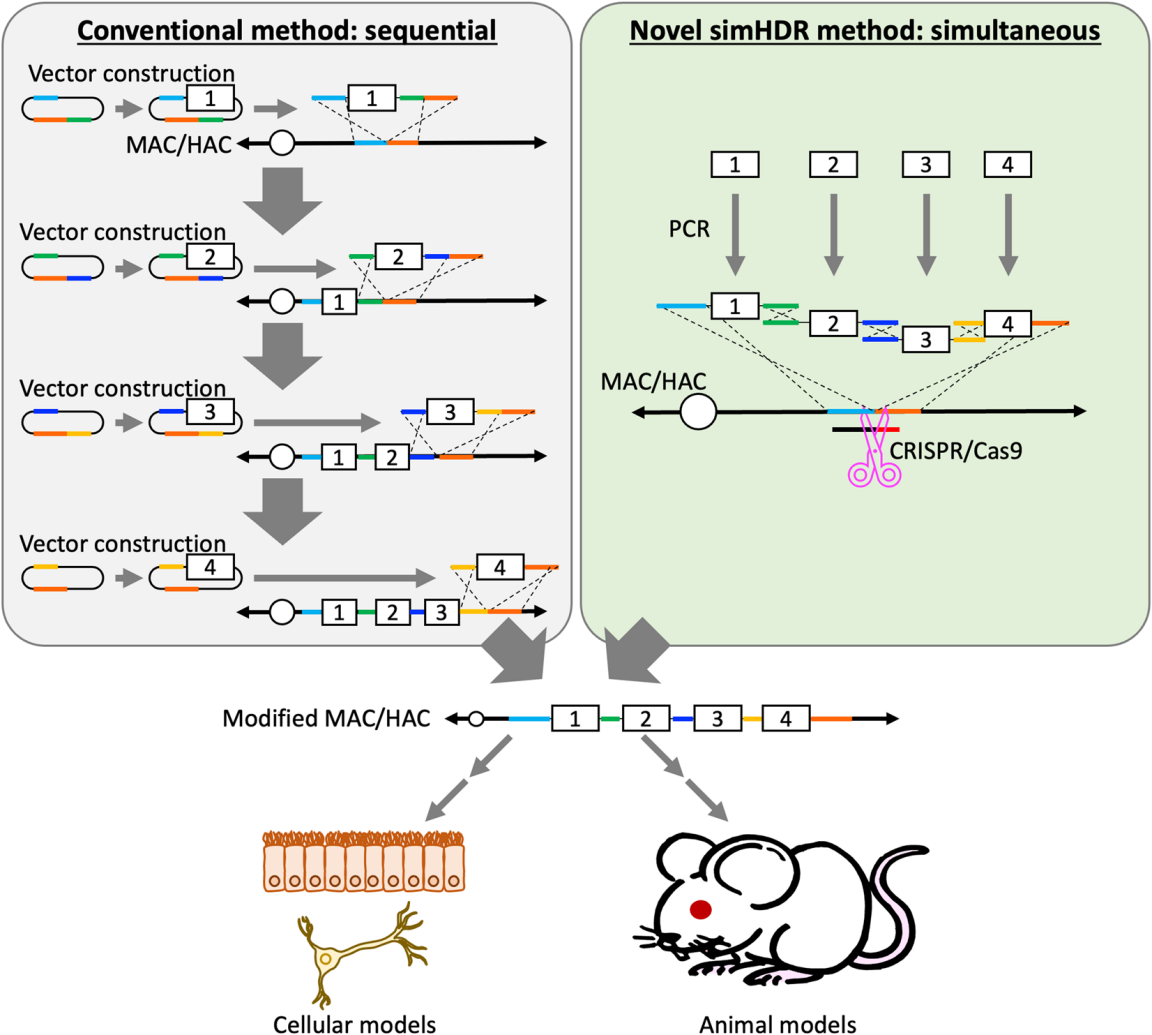
Schematic depiction of generating genetically modified models using MAC/HAC loaded with multiple HDR donors via conventional and novel methods. Conventional loading method of multiple HDR donors sequentially is shown in left. Novel loading method of multiple HDR donors simultaneously as reported in this study is shown in right. Constructed MAC/HAC contained multiple HDR donors that can subsequently be used for generating genetically modified cellular and animal models.

Previously, we have reported modification of mouse/human artificial chromosome vectors (MAC/HAC)^11,12^ in the DT40 cells showing high HDR efficiency, and application to generate genetically modified cells and animals^13–15^. MAC/HAC are generated from a native mouse/human chromosome by removing gene-cording regions and have unique features including stable independent maintenance from the host genome, distribution to daughter cells after cell division, and spatiotemporal expression dynamics according to the host expression control without silencing^11,16^. In other words, MAC/HAC has a safe harbor aspect and a significant advantage in generating genetically modified models. Furthermore, microcell-mediated chromosome transfer (MMCT) enables us to transfer MAC/HAC from DT40 cells to any cells^13,17–19^. By adding GOIs on MAC/HAC in the cells (donors) other than the ones (recipients) to be analyzed and subsequently transferring the MAC/HAC into the recipient cells by MMCT, it is possible to minimize concerns associated with unexpected mutations and insertions of GOIs into the recipient host genome. Previously, when generating designed MAC/HAC including the GOIs in the DT40 cells, we have loaded single GOI onto MAC/HAC by HDR on an individual basis^11,12^. This conventional homologous recombination type cloning requires multiple cloning and analysis steps (Fig. 1); therefore, the development of a technology that simultaneously loads multiple HDR donors would be of great value in the construction of modified MAC/HAC.

In this study, we report the simultaneous insertion of multiple fragments by HDR method termed simHDR, a technique wherein multiple HDR donors prepared by PCR can be loaded at one time onto MAC/HAC in the DT40 cells (Fig. 1). The high HDR efficacy of DT40 cells was utilized to introduce at least 4 HDR donors in a single step by using PCR products with homology arms (HAs). Furthermore, HA could be reduced to 60-bp which could be added to the primers. A conventional HDR donor is prepared through cloning of the GOI by adding the HA and selection markers, followed by transformation into *Escherichia coli*. To bypass these steps, we here attempted to prepare HDR donor fragments, GOI and selection markers by PCR. To understand gene functions including expression regulation, bacterial artificial chromosome (BAC) clones containing gene regulatory sequence are frequently used; however, available BAC clones are restricted offering limited number of genes with polymorphisms such as human leukocyte antigen (*HLA*). We confirmed that the simHDR could precisely load PCR-based HDR donor fragments onto MAC while maintaining functions of the *HLA-A* genomic region as a model case. Collectively, these results indicate that the simHDR can load multiple HDR donors simultaneously onto MAC. In addition, HDR donor fragments including the GOI and selection markers prepared by PCR can be directly loaded without HDR donor construction in *E. coli*.

## Results

### Improvement of HDR-KI efficacy by CRISPR/Cas9-mediated DSB in DT40 cells

Since CRISPR/Cas9-mediated KI is a highly efficient and specific targeting method in mammalian cells^20–23^, we applied CRISPR/Cas9-mediated KI method to the simHDR system. Previously, a novel MAC vector derived from mouse chromosome 10 (10MAC) was established and showed retention and stability comparable to 11MAC, which was used in both cells and animals as reported^24^. In this study, 10MAC2, one of the 10MAC series without EGFP and Blasticidin S (BS)-resistant genes, was used because EGFP and BS-resistance were used as selection markers loaded with fragments. Moreover, we designed sgRNA to target the 10MAC2 (Supplementary Fig. S1a) and constructed sgRNA-Cas9 all-in-one vector PX458.1a-MAC10CR1 that expressed the sgRNA to target the 10MAC2 and Cas9.

We first evaluated the efficiency of sgRNA for site-specific DSB in the DT40 cells after optimization of electroporation conditions using EGFP expression vector namely pCX-EGFP. The optimization was performed under the same conditions for the number of cells (1 × 10^6^ cells), buffer (Opti-MEM), and reaction volume (100 µL), and the conditions considered were voltage and DNA quantity. Based on these results, condition 7 (225 V, 10 µg) was used for the subsequent experiments (Supplementary Fig. S2). Then, we electroporated PX458.1a-MAC10CR1 and pCX-EGFP or only pCX-EGFP as Cas9 negative control group and collected EGFP positive cells by fluorescence-activated cell sorting (FACS). Cel-I assay was performed using genome DNA of the sorted cells to confirm that the sgRNA was recognized and Cas9 cleaved the target sequence^25^ (Supplementary Fig. S1b). The sgRNA designed in the previous studies was shown to be capable of DSB at the sgRNA-target sequence^22^.

Next, we tested whether CRISPR/Cas9-mediated DSB increases the efficiency of HDR-KI in the DT40 cells. For this, we utilized the HR1 plasmid vector designed to target the new left arm (nLA) and new right arm (nRA) on both sides of the sgRNA targeting site on 10MAC2 (Supplementary Fig. S1c). We analyzed the HR1 plasmid vector sequence focusing on nLA, EGFP, BS, TetR, and nRA (Supplementary Figs. S3–S8). We detected no mutations in the EGFP, BS, and TetR sequences. On the other hand, single nucleotide substitutions in the nLA and nRA sequences were detected. Since nLA and nRA sequences are homologous to the 10MAC2, we reasoned that these mutations would have no influence on the subsequent experiments. Then, we performed PCR to prepare HR1-BS containing EGFP and BS-resistant gene by using Pr.1/2 (Supplementary Fig. S1c and Table 1). We verified each HA sequence including HR1-BS fragment by sequencing, and no unexpected mutations were detected (Supplementary Figs. S3, S4, and S8). When HR1-BS is precisely recombined with nLA and nRA and loaded onto 10MAC2 by HDR, the DT40 cells become BS resistant and express EGFP (Supplementary Fig. S1c). We electroporated HR1-BS with or without PX458.1a-MAC10CR1 to DT40 cells containing 10MAC2 (DT40-10MAC2 cells) and selected with BS. To avoid contamination of the HR1 vector that was used as PCR template and reduce false positive clones such as random integration that could affect the results, 5-fluorocytosine (5-FC) selection was also performed to remove the cells with HR1 vectors containing Fcy::Fur. Then we obtained BS-resistant clones {HR1-BS with PX458.1a-MAC10CR1 [Cas9 (+)]: 14 and HR1-BS without PX458.1a-MAC10CR1 [Cas9 (−)]: 5} and EGFP-positive clones {HR1-BS with PX458.1a-MAC10CR1 [Cas9 (+)]: 13 and HR1-BS without PX458.1a-MAC10CR1 [Cas9 (-)]: 5} (Supplementary Fig. S1d). We next examined BS-resistant and EGFP-positive clones by PCR using junction primers to check the precise recombination. PCR-positive 11 clones [Cas9 (+)] (85% of the obtained EGFP-positive clones) and 3 clones [Cas9 (−)] (60% of the obtained EGFP-positive clones) were recombinants (Supplementary Fig. S1c, S1e and Table 1). These data indicated that CRISPR-Cas9-mediated DSB could increase HDR-KI efficiency in the DT40 cells like the other mammalian cell lines.

**Table 1:** Primer sequences.

Primer no	Primer name	Sequence (5′ > 3′)	References
Pr.1	nLA-F	AAATTAATTAACTGTGGCCCCCTATAACCATGA	
Pr.2	nRA-R2	GACCATGAAGATGGTCCAACTAAAGCAA	
Pr.3	Bsd-R	CTATGGCTTTGATCCCAGGATGCA	
Pr.4	Bsd-F	AGTGAACCGTCAGATCGCCTG	
Pr.5	EGFP500-R	AAAGAATTCTGCTCAGGTAGTGGTTGTCG	
Pr.6	EGFP-F	AAAGAATTCGCCACCATGGTGAGCA	
Pr.7	TetR-R	GTAGGTGTTTCCCTTTCTTCTTTAGCGA	
Pr.8	TetR-F	AAAGCTAGCGGTACCATGATGTCCAGAT	
Pr.9	EGFP2-F	TTGGCAAAAAACATGAATTCGCCACC	
Pr.10	3EGFP300-F	AAAGCGGCCGCACGGCAACTACAAGACCCGCG	
Pr.11	3EGFP60-F	AAAGCGGCCGCAAGCAGAACACCCCCATCGGC	
Pr.12	nLA60-F	AAGTGGGCATGACCTGAAATGTC	
Pr.13	nRA60-R	GAAGACTGCAGCAGATCCACTAG	
Pr.14	nLA-4kupA02	AAGTGGGCATGACCTGAAATGTCTCTAAAGAAATTAGAGGACCCAACCCCTTATCAAGAGACAAACCTGCACATCCTACACATGTACCCTGG	
Pr.15	3kdwnA02	GTAATTGATTACTATTAATAACTAGTCAATAATCAATGTCGACACGCGTGGTACCGGCCGATGTTTCTCTGCTGTGGACTCCATGGCCTTACTCC	
Pr.16	CAG-EGFP F	CGGCCGGTACCACGCGTGTCGACATTG	
Pr.17	Bsd-pA R	GAAGACTGCAGCAGATCCACTAGACACCAAGAGTCATCAGTACCAAGAGACCTACCAGATTCAATGTCAACGCGTGAATTCAGACATGAT	
Pr.18	KpnI_m10 LA F	TCGAGGTACCTCTAAGTCAGGGAAAGATCCCCTTCTTG	
Pr.19	nLA jun R	TCAATAATCAATGCACGCGTGGTACCGG	
Pr.20	HR1-EGFPjunc F	TTTGTCCCAAATCTGGCGGA	
Pr.21	HR1-EGFPjunc R	CAGCAGGGGGCTGTTTCATA	
Pr.22	HR1-TetRjunc F	GCCCTCTGGTTATGTGTGGG	
Pr.23	HR1-TetRjunc R	AGCTTTACACGCTTTGCTGC	
Pr.24	NheI-CmCherry-XbaI F	AAAGCTAGCACCATGGCCCTGAAGGGCGAGATCA	
Pr.25	neo310AS	GGTAGCCAACGCTATGTCCTGATAGCGGTC	
Pr.26	nLA-R	AAAGCGCGCCCTCTTGATAAGGGGTTGGGTCC	
Pr.27	nRA-F	AAAGGCGCGCCATCTGGTAGGTCTCTTGGTACT	
Pr.28	nRA-R	AAAGCGATCGCGACCATGAAGATGGTCCAACT	
Pr.29	CMVbsd-F	AAAGTCGACGAGGTCCGTTACATAACTTACGGTAA	
Pr.30	CMVbsd-R	CCCCGGGAATTCAGACATGATAAGA	
Pr.31	EGFP-R	AAAGATATCCCCGGGGTCGACCATGATTACG	
Pr.32	10NAC2-Cel1 F	AGGGGACATGGTCTGTGTGT	
Pr.33	10NAC2-Cel1 R	GGGGGTCTATGGGCAATCTG	
Pr.34	HLA-LAjun-R	GGAATTAAGCTGCACCTCTAGAAAGGAACA	
Pr.35	HLA-CAGjunF	GGTTCTAGCAACACCACATTCCACATCTAA	
Pr.36	CMVenh-R	AGTTTACCGTAAATAGTCCACCCATTGACG	
Pr.37	m10 crisp R	CTTCATCAGGACCCCAAACCTTTAAAGTCAAAGA	
Pr.38	bGH-pA F	CTGTGCCTTCTAGTTGCCAGCCATCTGTTG	
Pr.39	nLA-jun_SQ_F1	TGTCCCGAGGTCCACTATTC	
Pr.40	nLA-jun_SQ_R1	CCGTAAGTTATGTAACGCGG	
Pr.41	nLA60-jun_SQ_F1	ATCCACATCCTGCGAAAAAG	
Pr.42	nLA60-jun_SQ_R1	GTCCCCATGATCCAATCAAC	
Pr.43	CAG-jun_SQ_F1	GAAGCAGGATGGAGCTGAAC	
Pr.44	CAG-jun_SQ_R1	TGGGGAGAGTGAAGCAGAAC	
Pr.45	EGFP-jun_SQ_F1	ACGTAAACGGCCACAAGTTC	
Pr.46	EGFP-jun_SQ_R1	CTTGTACAGCTCGTCCATGC	
Pr.47	nRA60-jun_SQ_F1	TCCTGGGATCAAAGCCATAG	
Pr.48	nRA60-jun_SQ_R1	GCATTGGAGAGATTCGAAGG	
Pr.49	nLA-SQ-Rv2	AGTTGCCAGGTTGGATGCTG	
Pr.50	nRA-SQ-Fw2	AGTGGTGTCTTGGTTTCTGG	
Pr.51	nRA-SQ-Rv2	CACACCCAACCACTGCATTG	
Pr.52	cDNA HLA-A F2	TCCTTGGAGCTGTGATCACT	^2,9^
Pr.53	cDNA HLA-A R2	AAGGGCAGGAACAACTCTTG	^2,9^
Pr.54	DT40-GAPDH-F	GAGGGTAGTGAAGGCTGCTG	^3,6^
Pr.55	DT40-GAPDH-R	CATCAAAGGTGGAGGAATGG	^3,6^

### Simultaneous loading of more than 2 HDR donor fragments onto 10MAC2 in DT40 cells by simHDR

**Figure 2:**
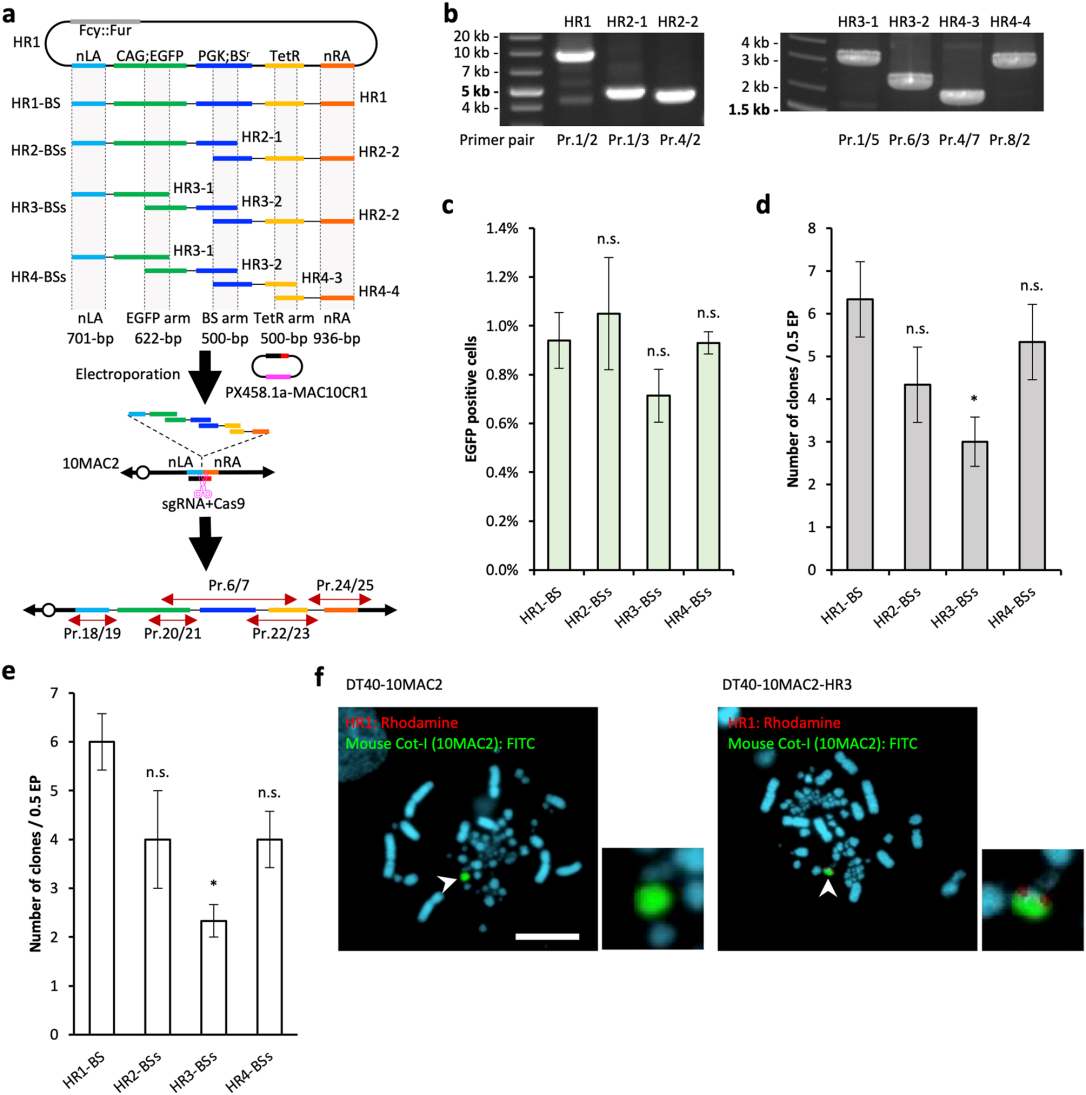
Assessment of the simHDR for 4 PCR HDR donor fragments. (**a**) Schematic representation of the simHDR of 1 to 4 PCR HDR donors loading onto the 10MAC2. Arrows indicate the position of PCR primers used for analysis. Dashed lines and gray shadings indicate homology arm position and length. (**b**) Preparation of PCR HDR donor fragments. Confirmation of precise amplification by electrophoresis. For gel source data, refer to Supplementary Fig. S13. (**c**) EGFP expression of DT40-10MAC2 cells containing recombined PCR-based HDR donors. The transient expression of EGFP was analyzed by FCM. Percentages of positive cells in each cell population are shown. (**d**) The BS-resistant and EGFP-positive clone number after BS selection. (**e**) Confirmation of precise simHDR by PCR analysis and the positive clone number are shown. (**f**) Representative image of metaphase FISH analysis with HR1 vector (red) and mouse Cot-I (green) detecting the 10MAC2. Arrowhead indicates the 10MAC2 and the inset shows an enlarged image thereof. Scale bar: 10 μm. (**c**)–(**e**), The data are expressed as means ± S.E. Statistical significance was determined by the Dunnett’s test. *P*-values of < 0.05 were considered significant and indicated by a single asterisk (∗). n.s.: not significant. Electroporation was performed independently for 3 times.

To examine the loading potential of more than 2 PCR HDR donor fragments by the simHDR, we performed PCR to prepare HDR donor fragments: HR1-BS; HR2-BSs (HR2-1 and HR2-2) that contained EGFP gene and split BS-resistant gene expression units to be non-functional themselves and contain overlap BS arms as a homologous sequence; HR3-BSs (HR3-1, HR3-2, and HR2-2) from HR1 vector, which contained BS arm same as HR2-BSs and split EGFP gene to be non-functional themselves and contain overlap EGFP arms as a homologous sequence; and HR4-BSs (HR3-1, HR3-2, HR4-3, and HR4-4) that contained BS arm, EGFP arm same as HR3-BSs, and TetR arm as a homologous sequence (Fig. 2a and b). We verified each HA sequence including PCR HDR donor fragments by sequencing, and no unexpected mutations were detected (Supplementary Figs. S3–S8). We electroporated HR1-BS, HR2-BSs, HR3-BSs, or HR4-BSs with PX458.1a-MAC10CR1 to DT40-10MAC2 cells following the electroporation condition as described in Supplementary Table S1. To confirm that the HDR donor fragments were transfected and recombined, we examined transient EGFP expression from recombinant DNA fragments with EGFP arm by flow cytometry (FCM) at 48 h after electroporation (Fig. 2c). After another 9 days of BS selection, we obtained BS-resistant clones (HR1-BS: 6.3, HR2-BSs: 4.3, HR3-BSs: 3.0, HR4-BSs: 5.3) and EGFP-positive clones (HR1-BS: 6.3, HR2-BSs: 4.0, HR3-BSs: 2.3, HR4-BSs: 4.3) (Fig. 2d and Table 2). We next examined the obtained clones by PCR using junction primers and confirmed that every condition contained precise recombinants (HR1-BS: 6.0, HR2-BSs: 4.0, HR3-BSs: 2.3, HR4-BSs: 4.0) (Fig. 2e and Table 2). The recombination efficiency was 1.2 × 10^–5^, 0.8 × 10^–5^, 0.5 × 10^–5^, and 0.8 × 10^–5^ for HR1-BS, HR2-BSs, HR3-BSs, and HR4-BSs, respectively (Table 2). Furthermore, we confirmed the presence of HR3-BSs-derived sequences on the 10MAC2 by fluorescence in situ hybridization (FISH). We detected colocalized signals of electroporated fragments and 10MAC2 (Fig. 2f). These results demonstrated that electroporated multiple PCR HDR donor fragments were successfully inserted into the targeted region on 10MAC2, not randomly on the chromosomes of DT40. Additionally, we verified the junction regions by sequencing, and no unexpected mutations were detected (Supplementary Figs. S3–S8). We noted a single base substitution in the nLA junction sequence in the HR1 vector; however, this substitution was not identified in the several simHDR products, which implied that this was not the result of the simHDR performance-based mutation. A similar case was also observed in the nRA sequence substitution. Taken together, these results clearly indicated that multiple HDR donor fragments prepared by PCR could be loaded onto MAC in a single step.

**Table 2:** Summary of obtained clone numbers and the simHDR efficiency related to the results shown in Fig. 2.

		HR1-BS	HR2-BSs	HR3-BSs	HR4-BSs
BS-resistant clones	1st	6	4	3	5
	2nd	5	3	2	4
	3rd	8	6	4	7
	Average	6.33	4.33	3.00	5.33
BS-resistant EGFP-positive clones	1st	6	3	2	4
	2nd	5	3	2	3
	3rd	8	6	3	6
	Average	6.33	4.00	2.33	4.33
(%)EGFP-positive/BS-resistant		100	92.3	77.8	81.3
Junction PCR positive clones	1st	6	3	2	4
	2nd	5	3	2	3
	3rd	7	6	3	5
	Average	6.00	4.00	2.33	4.00
(%)PCR-positive/EGFP-positive		94.7	100	100	92.3
Recombination efficiency		1.2 × 10^−5^	0.8 × 10^−5^	0.5 × 10^−5^	0.8 × 10^−5^

Next, to confirm that modified10MAC2 via the simHDR was also functional after its transfer to the other cells, we first performed MMCT of modified 10MAC2 to CHO cells (Supplementary Fig. S9a). We then transferred 10MAC2 constructed with HR3-BSs (10MAC2-HR3) from DT40 to CHO cells by the standard PEG-MMCT protocol as reported previously^26,27^. We obtained 32 BS-resistant and EGFP-positive CHO clones (Supplementary Fig. S9b). We next examined the 32 CHO clones by PCR using junction primers shown in Fig. 2a and Table 1 and confirmed that 30 clones had the 10MAC2-HR3. Furthermore, we confirmed the presence of the 10MAC2-HR3 independently in the CHO cells by FISH analysis. (Supplementary Fig. S9c). These results demonstrated that 10MAC2-HR3 was successfully transferred and maintained the gene functions after MMCT.

### Evaluation of homology arm lengths for the simHDR

**Figure 3:**
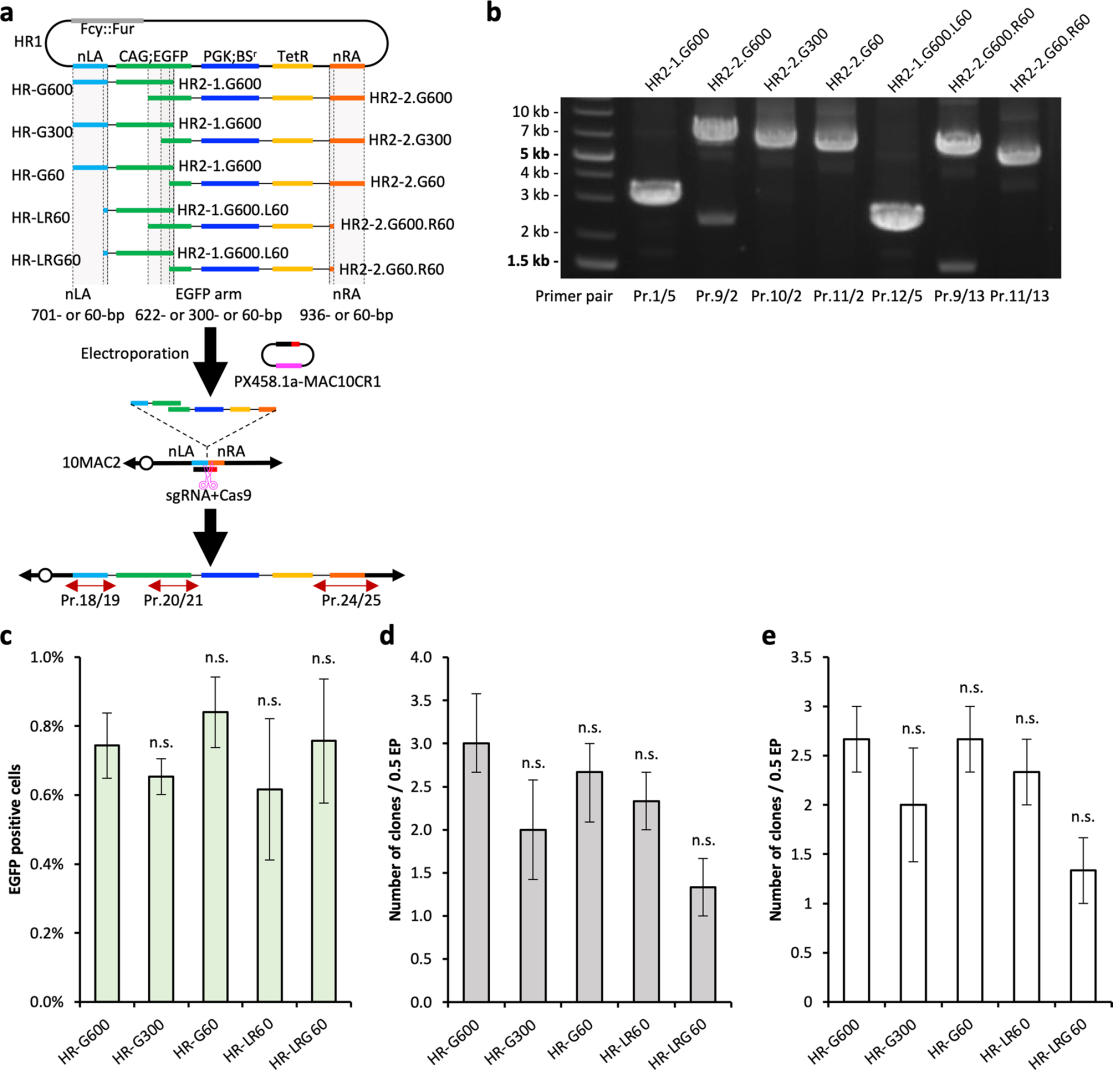
Comparison of homology arm lengths for the simHDR. (**a**) Schematic representation of the simHDR of each homology arm length PCR HDR donor loading onto the 10MAC2. Arrows indicate the position of PCR primers used for analysis. Dashed lines and gray shadings indicate homology arm position and length. (**b**) Preparation of PCR HDR donor fragments. Confirmation of precise amplification by electrophoresis. For gel source data, refer to Supplementary Fig. S14. (**c**) EGFP expression of DT40-10MAC2 cells containing recombined PCR-based HDR donors. The transient expression of EGFP was analyzed by FCM. Percentages of positive cells in each cell population are shown. (**d**) The BS-resistant and EGFP-positive clone number after BS selection. (**e**) Confirmation of precise simHDR by PCR analysis and the positive clone number are shown. (**c**)–(**e**) The data are expressed as means ± S.E. Statistical significance was determined by the Dunnett’s test. *P*-values of < 0.05 were considered significant. n.s., not significant. Electroporation was performed independently for 3 times.

To explore the efficacy of the simHDR insertion depending on HA lengths, we produced PCR HDR donor fragments with a variety of HA lengths (60-, 300- and 622-bp). We performed PCR to prepare HR-G60 (HR2-1.G600 and HR2-2.G60), HR-G300 (HR2-1.G600 and HR2-2.G300), HR-G600 (HR2-1.G600 and HR2-2.G600), HR-LR60 (HR2-1.G600.L60 and HR2-2.G600.R60), and HR-LRG60 (HR2-1.G600.L60 and HR2-2.G60.R60) (Fig. 3a and b). We verified the HA sequence including PCR HDR donor fragments by sequencing, and no unexpected mutations were detected (Supplementary Fig. S10). We electroporated these fragments with PX458.1a-MAC10CR1 to DT40-10MAC2 cells as described in Supplementary Table S2 and confirmed transient EGFP expression from recombinant DNA HDR donor fragments with EGFP arm by FCM at 48 h after electroporation (Fig. 3c). After another 9 days of BS selection, BS-resistant clones (HR-G600: 3.3, HR-G300: 3.0, HR-G60: 5.0, HR-LR60: 2.7, HR-LRG60: 2.7) and EGFP-positive clones (HR-G600: 3.0, HR-G300: 2.0, HR-G60: 2.7, HR-LR60: 2.3, HR-LRG60: 1.3) were obtained (Fig. 3d and Table 3). We next examined the obtained clones by PCR using junction primers and confirmed every condition contained expected recombinants (HR-G600: 2.7, HR-G300: 2.0, HR-G60: 2.7, HR-LR60: 2.3, HR-LRG60: 1.3) (Fig. 3e and Table 3). The efficiency of recombination was 5.3 × 10^–6^, 4.0 × 10^–6^, 5.3 × 10^–6^, 4.7 × 10^–6^, and 2.7 × 10^–6^ for HR-G600, HR-G300, HR-G60, HR-LR60, and HR-LRG60, respectively (Table 3). Additionally, we verified the junction regions of HR-LRG60 clones by sequencing, and no unexpected mutations were detected (Supplementary Fig. S10). These data indicate that 60-bp of HAs for the simHDR is sufficient; importantly, there is an upper limit to the sequence length that can be added to a PCR primer, but 60-bp is technically feasible. This suggests that HDR donor fragments can be prepared directly by PCR without any cloning and recombination into *E. coli* to add HAs to the sequences to be cloned into the MAC and selection markers.

**Table 3:** Summary of obtained clone numbers and the simHDR efficiency related the results shown in Fig. 3.

		HR-G600	HR-G300	HR-G60	HR-LR60	HR-LRG60
BS-resistant clones	1st	3	2	4	2	3
	2nd	4	4	6	3	3
	3rd	3	3	5	3	2
	Average	3.33	3.00	5.00	2.67	2.67
BS-resistant EGFP-positive clones	1st	3	1	2	2	1
	2nd	4	3	3	2	2
	3rd	2	2	3	3	1
	Average	3.00	2.00	2.67	2.33	1.33
(%)EGFP-positive/BS-resistant		90.0	66.7	53.3	87.5	50.0
Junction PCR positive clones	1st	3	1	2	2	1
	2nd	3	3	3	2	2
	3rd	2	2	3	3	1
	Average	2.67	2.00	2.67	2.33	1.33
(%)PCR-positive/EGFP-positive		88.9	100	100	100	100
Recombination efficiency		5.3 × 10^−6^	4.0 × 10^−6^	5.3 × 10^−6^	4.7 × 10^−6^	2.7 × 10^−6^

### Direct loading of PCR product from culture cells onto 10MAC2 by the simHDR

In homologous recombination type cloning, cloning the GOI, selection markers and HR arms into a plasmid vector or modifying BAC/PAC has been indispensable^28^. However, there are a few critical issues; for example, cloning of the fragments into a plasmid vector is needed, and in the case of a polymorphic gene, the available BAC/PAC offers limited variations in polymorphisms. Since our experiments showed that multiple PCR fragments with a 60-bp HA could be loaded at once (Fig. 3), the simHDR has the possibility of preparing the GOI of target individual and the selection markers by PCR and loading them onto the MAC. Therefore, we attempted to directly insert the target gene to 10MAC2 without cloning into a plasmid vector by combining PCR and the simHDR using genomic DNA obtained from cells with the target gene as a HDR donor template.

**Figure 4:**
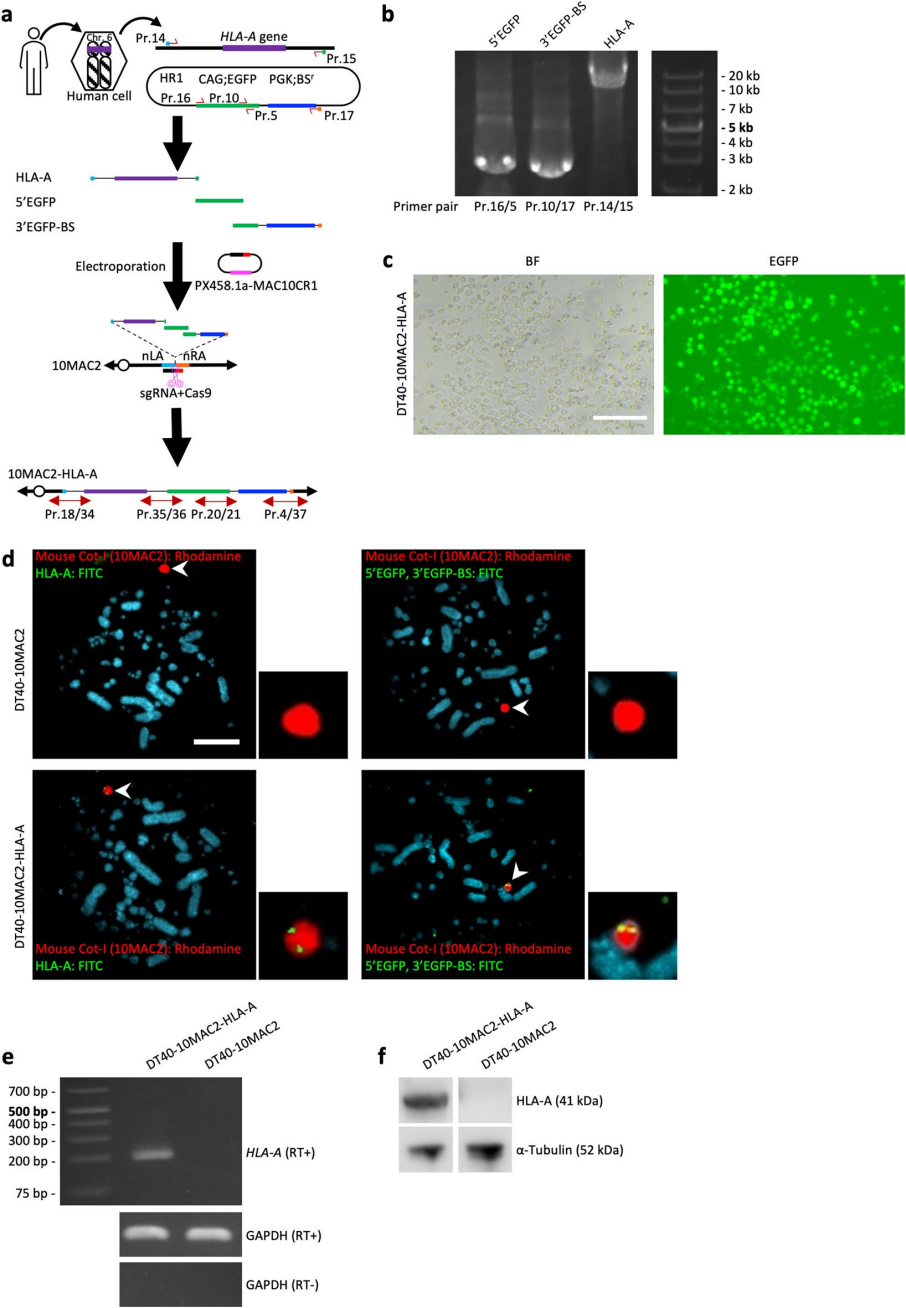
The simHDR based direct cloning of *HLA-A* genomic region. (**a**) Schematic representation of the simHDR illustrating the loading of genomic DNA sequence from human cells onto the 10MAC2. Arrows indicate the position of PCR primers used for analysis. (**b**) Preparation of PCR HDR donor fragments. Confirmation of precise amplification by electrophoresis. For gel source data, refer to Supplementary Fig. S15. (**c**) Image of DT40 cells carrying the 10NAC2-HLA-A. EGFP expression indicates the presence of the 10NAC2-HLA-A. BF, bright field. Scale bar: 100 µm. (**d**) Representative image of metaphase FISH analysis with mouse Cot-I (red) detecting 10MAC2 and HLA-A or 5’EGFP and 3’EGFP-BS PCR HDR donor fragments (green). Arrowhead indicates the 10MAC2 and the inset shows an enlarged image thereof. Scale bar: 10 μm. (**e**) RT-PCR products generated from DT40-10MAC2-HLA-A and DT40-10MAC2 cells cDNAs. Each end point PCR products are shown. For gel source data, refer to Supplementary Fig. S16. (**f**) Detecting HLA-A protein by western blotting. For membrane source data, refer to Supplementary Fig. S17.

We examined the loading of *HLA-A* gene isolated from HepG2, a human hepatoma cell line onto 10MAC2 by the simHDR method (Fig. 4a). The 10-kb genomic DNA containing *HLA-A* gene and 5'- and 3'-UTRs required for its expression regulation was extracted from HepG2. The primer used for PCR was adapted for the simHDR to target *HLA-A* genomic region loading by adding a 60-bp HA for nLA and a 60-bp HA for CAG-EGFP (Fig. 4a and b). To detect the loading of the HLA-A fragment onto 10MAC2, additional fragments were prepared to allow the BS and EGFP selections. HR1 was used as a template to create 5' EGFP and 3' EGFP-BS fragments which were prepared with the primer producing a 300-bp EGFP arm and a 60-bp HA for nRA (Fig. 4a and b). We note that we used 300-bp EGFP arm because PCR for 60-bp EGFP arm could not be amplified as a single product. Similarly, we can choose the optimal primer in the range of 60- to 622-bp EGFP arms. We verified each HA sequence including PCR HDR donor fragments by sequencing, and no unexpected mutations were detected (Supplementary Fig. S11). We electroporated these fragments with PX458.1a-MAC10CR1 to DT40-10MAC2 cells as described in Supplementary Table S3 and obtained 12 BS-resistant and EGFP-positive clones (Fig. 4c). We next examined the 12 clones by PCR using junction primers and confirmed 2 clones. Furthermore, we confirmed the presence of HLA-A fragment sequence on the 10MAC2 by FISH. We detected colocalized signals of electroporated fragments and 10MAC2 (Fig. 4d). Additionally, we verified the junction regions by sequencing, and no unexpected mutations were detected (Supplementary Fig. S11). These results indicated that the electroporated *HLA-A* genomic region from HepG2 was successfully inserted into the targeted region on 10MAC2 directly, not randomly on the chromosomes of DT40.

Next, to analyze the functionality of the loaded *HLA-A* region, mRNA transcription was examined by RT-PCR using primers detecting from exon 5 to 3’UTR as described by Laura et al.^29^. Consistent with transcription of the *HLA-A* gene, we were able to amplify PCR products at the expected size, but not from the negative control (DT40-10MAC2 cells) (Fig. 4e). Finally, to determine whether *HLA-A* transcripts were translated, we examined HLA-A proteins by western blotting. We harvested total cell protein, and identified bands indicative of full- length HLA-A from DT40-10MAC2-HLA-A total cell lysate, but not from DT40-10MAC2 total cell lysate (Fig. 4f). These data indicated that the *HLA-A* genomic region was successfully loaded onto 10MAC2 by the simHDR, as well as transcribed and translated as a full-length protein.

## Discussion

In this study, we developed a novel multiple DNA loading method termed simHDR for MAC in the DT40 cells. The simHDR requires only PCR HDR donor fragments containing HAs without cloning to plasmid vectors and transformation into *E. coli*. In conventional methods for constructing designed MACs, it was necessary to modify the target plasmid vector to incorporate homologous recombination type cloning^11,12^. In addition, homologous recombination type cloning requires screening and analysis each time a single HDR donor is loaded, and since the number of drugs that can be used to load genes is limited, drug-resistant genes must be knocked out to continuously load HDR donors, resulting in the laborious effort. Therefore, the development of a technology that simultaneously loads multiple HDR donors in the DT40 cells would be of great value in the construction of designed MAC/HAC.

The simHDR was shown to be capable of loading at least HR4-BSs built up by four fragments onto 10MAC2 (Fig. 2). Surprisingly, between HR1- to HR3-BSs, the number of clones obtained decreased as the number of fragments increased, and at HR4-BSs, the number of clones obtained was exceptionally comparable to that of HR2-BSs. This was contrary to the expectation that the number of clones obtained would decrease as the number of fragments increased, since the probability of introducing all fragments into the cell decreases as the number of fragments increase. In this experimental design, the sequences constructed on the MAC are identical under all conditions, and the length of each fragment becomes shorter as the number of fragments increase. Considering that the efficiency of DNA introduction into cells and cell viability are inversely proportional to DNA size^30^, and that an increase in the number of fragment types decreases the probability that all types of donor fragments will be introduced into cells simultaneously, the number of fragment types and the size of each fragment is an important parameter to design. Thus, it is an important future consideration for the simHDR to examine optimized preparation of the size and number of PCR HDR donor fragments.

The use of the HDR-KI efficiency of DT40 cells is also important for the development of the simHDR. In mouse ES cells, which have the highest HDR-KI efficiency among the mammalian cells, the HDR-KI efficiency is approximately 0.5%, and 4–15% when combined with CRISPR/Cas9-mediated DSB^5,10^. On the other hand, in the DT40 cells, HDR-KI efficiency is as high as 60%, and the efficiency increases to 85% when combined with CRISPR/Cas9-mediated DSB (Supplementary Fig. S1). In addition, the use of selection markers as HAs in the simHDR successfully raised HDR-KI efficiency to 90–100% (Fig. 2 and Table 2). For the simHDR to effectively acquire the desired clones, the approach of reconstructing selection markers by HDR is potentially significant. Reconstruction of selection markers increased the ratio of PCR-positive clones to EGFP-positive clones compared to the ratio of the clones without reconstruction (Fig. 2 and Table 2). This is due to the presumption that BS-resistant and EGFP-positive clones included the randomly integrated clones in conditions such as the HR1-BS that does not require reconstruction of the selection markers. The conventional HDR-KI method also has the problem of random integration because target GOIs and selection markers are cloned into a single vector. The ability to reduce these issues is also an important advantage of the simHDR method.

HDR-KI requires HAs that are designed to target region, and HA length affects the accuracy of HDR-KI in the mouse ES cells, which have the high HDR efficiency among mammalian cells^10^. Therefore, HAs of mostly 1 to several kb are often used in HDR-KI. We evaluated the HA lengths of the simHDR in the DT40 cells (Fig. 3 and Table 3). Surprisingly, shortening HA length to 60-bp did not affect HDR-KI efficiency of the simHDR in the DT40 cells as evidenced by the accuracy of 60-bp HAs for the reconstruction of functional EGFP gene. Since 60-bp HAs for each fragment can be added to PCR primers, preparation of HDR donor fragments does not require cloning and recombination into *E. coli* to add HAs to target sequences and selection markers. In all, HDR donor fragments can be prepared directly by only PCR.

To explore the broader application of the simHDR, we attempted to clone genomic regions amplified by PCR from the genomic DNA of cells directly into MAC. In previous studies, BAC clones have been frequently used for the generation of cellular and animal models^31,32^. However, BAC clones not only do not cover all species but are also restricted to clones derived from limited individuals within a species^33^. Hence, the BAC is not appropriate for generating models of polymorphic genes like *HLA*. To overcome this problem, we cloned the 10-kb *HLA-A* genomic region from human cells as a model case for genomic region cloning by the simHDR. The 10-kb HLA-A fragment was prepared by PCR using genomic DNA and subsequently loaded onto the MAC (Fig. 4). Furthermore, the loaded *HLA-A* was successfully expressed as demonstrated by RT-PCR and western blotting. These results indicate that the genomic region amplified by PCR using desired human cell DNA template can be cloned directly into the MAC by the simHDR.

When the simHDR system is used for functional analysis of genes and therapeutic application, quality control is important to verify that the final product has no mutations. Therefore, it is essential to eliminate the possibility of unexpected mutation/insertion in the final product by ensuring the intact whole genome sequence. The frequency of mutations in the final product involves the accuracy of the PCR, simHDR, and MMCT steps. To confirm the accuracy of simHDR, we compared the sequences of the PCR HDR donor fragments before loading with the 10MAC2 sequence after loading. We specifically focused our sequence analysis on/around the HAs because mutations during HDR are occasionally detected on/around HAs, and found no unexpected mutations. Since HDR is a highly accurate repair pathway, the simHDR using HDR contributes to reduction of the mutation rate in the simHDR products. Furthermore, mutations and chromosomal rearrangements that occur during PCR or MMCT are problematic; hence, analyzing the sequences of PCR HDR donor fragments, simHDR products and post-MMCT clones to eliminate those with mutations is important in obtaining a quality-controlled final product for functional analysis of genes and therapeutic application.

Generation of genetically modified cellular and animal models play a pivotal role in the research of gene functions and in understanding the mechanisms of diseases. The novel simHDR method described herein is a rapid and convenient approach to achieve this goal. In addition, the preparation of HDR donor fragments via PCR contributes to generating more flexible design of cellular and animal models. Our strategy has the potential not only to be used to generate cellular and animal models but also to be broadly adapted to experiments for rapid and stable cloning of viral genomes and immunogens. Taken together, the gene cloning system onto MAC/HAC in the DT40 cells via simHDR described in this study will be broadly used to generate models, investigate gene functions, and understand disease mechanisms and therapeutic interventions.

## Material and method

### Vector construction and PCR HDR donor fragment preparation

For targeting of 10MAC2, we constructed HR1 vector (Supplementary Fig. S1c). The two HAs, nLA and nRA, BS resistant gene, and EGFP gene were amplified by PCR using primers, Pr.1/26, Pr.27/28, Pr.29/30, and Pr.6/31 (Table 1), respectively, and digested with PacI/AscI, PacI/AsiSI, SalI/EcoRI, and AsiSI (NEB, MA, USA), respectively. The sequence including TetR arm was prepared from pcDNA3.1-TetR-NLS-linker-mCherry by digesting with MluI/PsiI (NEB). Each digested fragment was ligated sequentially.

The CRISPR/Cas9 targeting sequence for 10MAC2 was 5′-CAACCCCTTATCAAGAGATC-3′ (Supplementary Fig. S1a). PX458a vector encoding the improved gRNA scaffold sequence was utilized for construction of sgRNA-Cas9 all-in-one vector PX458.1a-MAC10CR^34,35^. PX458 is a gift from Feng Zhang (Addgene plasmid #48138; http://n2t.net/addgene:48138; RRID: Addgene 48138).

HR1, HR2-1, HR2-2, HR3-1, HR3-2, HR4-3, and HR4-4 were amplified by PCR using primers, Pr.1/2, Pr1/3, Pr.4/2, Pr.1/5, Pr.6/3, Pr.4/7, and Pr.8/2, respectively (Fig. 2 and Table 1). HR2-1.G600, HR2-2.G600, HR2-2.G300, HR2-2.G60, HR2-1.G600.L60, HR2-2.G600.R60, and HR2-2.G60.R60 were amplified by PCR using primers, Pr.1/5, Pr.9/2, Pr.10/2, Pr.11/2, Pr.12/5, Pr.9/13, and Pr.11/13, respectively (Fig. 3 and Table 1). HLA-A, 5’EGFP, and 3’EGFP-BS were amplified by PCR using primers, Pr.14/15, Pr.16/5, and Pr.10/17, respectively (Fig. 4 and Table 1). PCR was performed using KOD FX (Toyobo, Osaka, Japan) or KOD One PCR Master Mix -Blue- (Toyobo). PCR products were purified with QIAquick PCR Purification Kit (QIAGEN, Hilden, Germany) and MonoFas DNA Purification Kit I (ANIMOS, Gunma, Japan) and concentrated by isopropanol precipitation following the standard method.

### Cell culture

DT40 cells (RCB1464) were purchased and obtained with permission from RIKEN BRC, Ibaraki, Japan. The cells were maintained at 40 °C at 10% CO_2_ in Roswell Park Memorial Institute (RPMI) medium 1640 (Fujifilm-Wako, Osaka, Japan) supplemented with 10% fetal bovine serum (FBS; Sigma-Aldrich, MO, US), 1% chicken serum (Gibco, Thermo Fisher Scientific, MA, USA), 1% penicillin–streptomycin solution (× 100) (PS; Fujifilm-Wako) and 50 µM 2-mercaptoethanol (2ME; Thermo Fisher Scientific, MA, USA). DT40 cells containing 10MAC2 (DT40-10MAC2 cells) were generated previously^24^. We initially examined the sensitivity of DT40-10MAC2 cells to the selection medium. The DT40-10MAC2 cells were sensitive to 15 µg/mL of blasticidin S hydrochloride (BS; Funakoshi, Tokyo, Japan) and 250 µg/mL of 5-fluorocytosine (5-FC; InvivoGen, CA, USA). We found no obvious living cells after 7 days of selection. Hprt-deficient CHO cells (CHO (*Hprt*^−/−^); JCRB0218) were purchased and obtained with permission from NIBIOHN, Osaka, Japan. The cells were maintained at 37 °C at 5% CO_2_ in Ham's F-12 (Fujifilm-Wako) supplemented with 10% FBS and 1% PS. HepG2 cells (RCB1886) were purchased and obtained with permission from RIKEN BRC. The cells were maintained at 37 °C at 5% CO_2_ in Dulbecco’s modified Eagle medium (DMEM; Fujifilm-Wako) supplemented with 10% FBS and 1% PS. No human participants and animals were involved in this study.

### Electroporation

Before electroporation, 1 × 10^6^ cells of DT40-10MAC2 cells were washed once with serum-free DMEM and resuspended in Opti-MEM (Thermo Fisher scientific). Each PCR HDR donor fragment was added to 100 µL of cell suspension, mixed, and electroporated using SuperElectroporator NEPA21 (NEPA GENE, Chiba, Japan) (Supplementary Tables S1–S3). Following the electroporation, the cells were diluted with DT40 culture medium and plated onto 6-well plates. After 24 h of incubation in a nonselective DT40 culture medium, half of the cells from 6-well plates were passed to the 96-well plates with step dilution (1/2, 1/4, 1/8, 1/16, 1/32, 1/64, and 1/128). After another 24 h of incubation in the DT40 culture medium, DT40 culture medium was supplemented with 15 µg/mL of BS. The cells were maintained under BS selection for 5 days, followed by additional 3 days of selection in 15 µg/mL of BS and 250 µg/mL of 5-FC. At the end of the incubation period, the surviving cells were passed to 6-well plates and cloned. Recombinants were identified by PCR and FISH analysis. Electroporation was replicated three independent times.

To detect transient EGFP expression from recombinant PCR HDR donor fragments with EGFP arm, we performed FCM with CytoFlex S (Beckman Coulter, CA, USA). At 48 h after electroporation of PCR HDR donor fragments, half of DT40-10MAC2 cells grown in 6-well plate was used for FCM analysis, and EGFP-positive cells were detected using the FITC channel.

### Cel-I assay to detect DSB

Before Cel-I assay, 10 µg of PX458.1a-MAC10CR1 and 0.6 µg of pCX-EGFP were electroporated into 1 × 10^6^ DT40-10MAC2 cells and plated onto 6-well plate. At 48 h after incubation, FACS was performed with a MoFlo XDP (Beckman Coulter), and 9.08 × 10^4^ EGFP-positive cells were harvested in 10 cm dish. After another 24 h, genomic DNA of DT40-10MAC2 cells were extracted using the standard method and step-down PCR was performed using TaKaRa Ex Taq (Takara, Shiga, Japan) and primers Pr.32/33 (Table 1). Cel-I assay was performed using Surveyor Mutation Detection Kit (Catalog No. 706025; IDT, NJ, USA)　according to manufacturer’s protocol. Products were then resolved by electrophoresis on agarose gels followed by staining with ethidium bromide. GeneRuler 1 kb Plus DNA Ladder (Thermo Fisher Scientific) was used as a band size marker. Positive control was prepared by PCR using control G and C plasmids contained in Surveyor Mutation Detection Kit following the manufacturer’s protocol.

### PCR analysis

Genomic DNA of the cells were extracted using the standard method and PCR was performed for nLA-, EGFP-, BS-, TetR-, and nRA-junction by using primers, Pr.18/19, Pr.20/21, Pr.6/7, Pr.22/23, and Pr.24/25, respectively (Figs. 2a, 3a, Supplementary Fig. S1c, and Table 1) and for nLA2-, CAG-, EGFP-, and nRA2-junction by using primers, Pr.18/34, Pr.35/36, Pr.20/21, and Pr.4/37, respectively (Fig. 4a and Table 1). PCR was performed using KOD FX or KOD One PCR Master Mix -Blue-. PCR products were then resolved by electrophoresis on agarose gels followed by staining with ethidium bromide. GeneRuler 1 kb Plus DNA Ladder was used as a band size marker.

### Fluorescence in situ hybridization (FISH) analysis

Before FISH analysis, DT40 cells were incubated for 15 min in the DT40 culture medium supplemented with 0.05 µg/mL of Colcemid or CHO cells were incubated for 1 h in the CHO culture medium supplemented with 0.1 µg/mL of Colcemid, harvested, incubated for 15 min in 0.075 M KCl, and fixed with methanol and acetic acid (3:1). The slides were prepared using the standard method. FISH analysis was performed using fixed metaphase spreads of each cell hybrid using digoxigenin-labeled (Roche, Basel, Switzerland) mouse Cot-I DNA (Invitrogen), and biotin-labeled HR1 vector or PCR HDR donors as described previously^14^. Chromosomal DNA was counterstained with 4,6-diamidino-2-phenylindole (DAPI; Sigma-Aldrich). Images were captured using an AxioImagerZ2 fluorescence microscope (Carl Zeiss GmbH, Jena, Germany).

### Microcell-mediated chromosome transfer (MMCT)

MMCT was performed as described previously^27^. The 10MAC2-HR3 cells were transferred to CHO (*Hprt*^−/−^) cells. The CHO cells fused with DT40(10MAC2-HR3)-derived microcells were selected with 8 µg/mL of BS and ouabain octahydrate (Sigma-Aldrich), then we obtained BS-resistant and EGFP-positive clones. The clones were identified by PCR and FISH analysis.

### Sequencing analysis

To verify the sequences of HR1 plasmid vector as PCR template, PCR HDR donor fragments, and modified 10MAC2 constructed by simHDR, each target region was amplified by PCR and directly sequenced using internal primers. The primers used for PCR amplification and sequencing are described in the Supplementary Figs. S4–S8, S10, and S11, and Table 1. The analyzed sequences were aligned using MUSCLE.

### Reverse transcription PCR (RT-PCR)

The DT40-10MAC2 and DT40-10MAC2-HLA-A cells were grown to 80% confluency in 6 cm dishes, total RNA was isolated with RNeasy Plus Mini Kit (QIAGEN), and cDNA was synthesized from total RNA with SuperScript IV Reverse Transcriptase (Thermo Fisher Scientific). Control RT reactions were otherwise processed identically, except for the omission of reverse transcriptase from the reaction mixture. Finally, PCR was carried out with QuantiTect SYBR Green PCR Kits (QIAGEN) with 95 °C/10 s, 60 °C/60 s. Primers used for HLA-A was Pr.52/53 as described by Laura et al.^29^ (Table 1). Other primers used were Pr.54/55 for avian GAPDH as described by Rong et al.^36^ (Table 1). Each PCR products were resolved by electrophoresis on agarose gels followed by staining with ethidium bromide. GeneRuler 1 kb Plus DNA Ladder was used as a band size marker.

### Western blotting

The DT40-10MAC2 and DT40-10MAC2-HLA-A cells were grown to 80% confluency in 6-well plates, and the cells were rinsed in cold PBS, then lysed in 2 × Laemmli Sample Buffer (BIO-RAD, CA, USA) supplemented with 2ME. The lysates were sonicated with BioruptorPlus (Diagenode, Liège, Belgium) for 10 min (on: 30 s, off: 30 s at low level), then boiled. The lysates were separated via SDS-PAGE on NuPAGE 4–12% Bis–Tris Gel (Thermo Fisher Scientific) for 42 min at 200 V with PowerEase Touch 350 W Power Supply (Thermo Fisher Scientific), then transferred to Invitron PVDF Filter Paper Sandwich (0.45 µm Pore Size, Thermo Fisher Scientific) for 1 h at 20 V with PowerEase Touch 350 W Power Supply. The membranes were blocked for 1 h in 5% nonfat dry milk (CST, MO, USA) in PBST (0.1% Tween 20 in PBS) and incubated overnight at 4 °C with rabbit monoclonal anti-HLA-A antibody (ab52922; 1/10,000; Abcam, Cambridge, UK) or incubated for 1 h at RT with rabbit monoclonal anti-α-tubulin antibody (#2125; 1/10,000; CTS) in 5% nonfat dry milk in PBST. The membranes were washed 3 times in PBST, incubated with goat anti-rabbit IgG secondary antibody H&L (HRP) (ab205718; 1/10,000; Abcam) for 1 h at RT, and washed 3 times in PBST. Protein bands were visualized via SuperSignal West Femto Maximum Sensitivity Substrate (Thermo Fisher Scientific) for HLA-A membrane and Pierce ECL Western Blotting Substrate (Thermo Fisher Scientific) for α-tubulin membrane. Images were acquired by using ImageQuant LAS4000 (GE Healthcare, IL, USA). Precision Plus Protein Standards Dual Color (BIO-RAD) was used as a band size marker.

### Statistical analysis

Statistical significance was determined by the Dunnett’s test. *P*-values of < 0.05 were considered significant and indicated by a single asterisk (∗).

## Supplementary Information

Supplementary Information.

## Data Availability

All data generated or analyzed during this study are included in this published article [and its supplementary information files].
