# Publisher Correction: Simultaneous loading of PCR-based multiple fragments on mouse artificial chromosome vectors in DT40 cell for gene delivery

**DOI:** 10.1038/s41598-023-30359-8

**Published:** 2023-02-28

**Authors:** Kyotaro Yamazaki, Kyosuke Matsuo, Akane Okada, Narumi Uno, Teruhiko Suzuki, Satoshi Abe, Shusei Hamamichi, Nanami Kishima, Shota Togai, Kazuma Tomizuka, Yasuhiro Kazuki

**Affiliations:** 1 Department of Chromosome Biomedical Engineering, Integrated Medical Sciences, Graduate School of Medical Sciences, Tottori University, 86 Nishi-Cho, Yonago, Tottori 683-8503 Japan; 2 Department of Biomedical Science, Institute of Regenerative Medicine and Biofunction, Graduate School of Medical Sciences, Tottori University, 86 Nishi-Cho, Yonago, Tottori 683-8503 Japan; 3 Chromosome Engineering Research Center, Tottori University, 86 Nishi-Cho, Yonago, Tottori 683-8503 Japan; 4 Laboratory of Bioengineering, Faculty of Life Sciences, Tokyo University of Pharmacy and Life Sciences, 1432-1 Horinouchi, Hachioji, Tokyo 192-0392 Japan; 5 Stem Cell Project, Tokyo Metropolitan Institute of Medical Science, Kamikitazawa, Setagaya-Ku, Tokyo 156-8506 Japan; 6 Department of Chromosome Biomedical Engineering, Institute of Regenerative Medicine and Biofunction, Graduate School of Medical Sciences, Tottori University, 86 Nishi-Cho, Yonago, Tottori 683-8503 Japan; 7 Department of Chromosome Biomedical Engineering, School of Life Science, Faculty of Medicine, Tottori University, 86 Nishi-Cho, Yonago, Tottori 683-8503 Japan; 8 Chromosome Engineering Research Group, The Exploratory Research Center on Life and Living Systems (ExCELLS), National Institutes of Natural Sciences, 5-1 Higashiyama, Myodaiji, Okazaki, Aichi 444-8787 Japan

Correction to: *Scientific Reports* 10.1038/s41598-022-25959-9, published online 16 December 2022

The original version of this Article contained errors in Figures 1 and 4, as well as in the Supplementary Information.

In the left panel of Figure 1, the colour of the line to the left of the white box with the number “3” shown in the diagram of the conventional method was incorrect. In addition, the text “Vector construction” has been added to each step in the diagram of the conventional method. The original Figure 1 and accompanying legend appear below.

**Figure 1:**
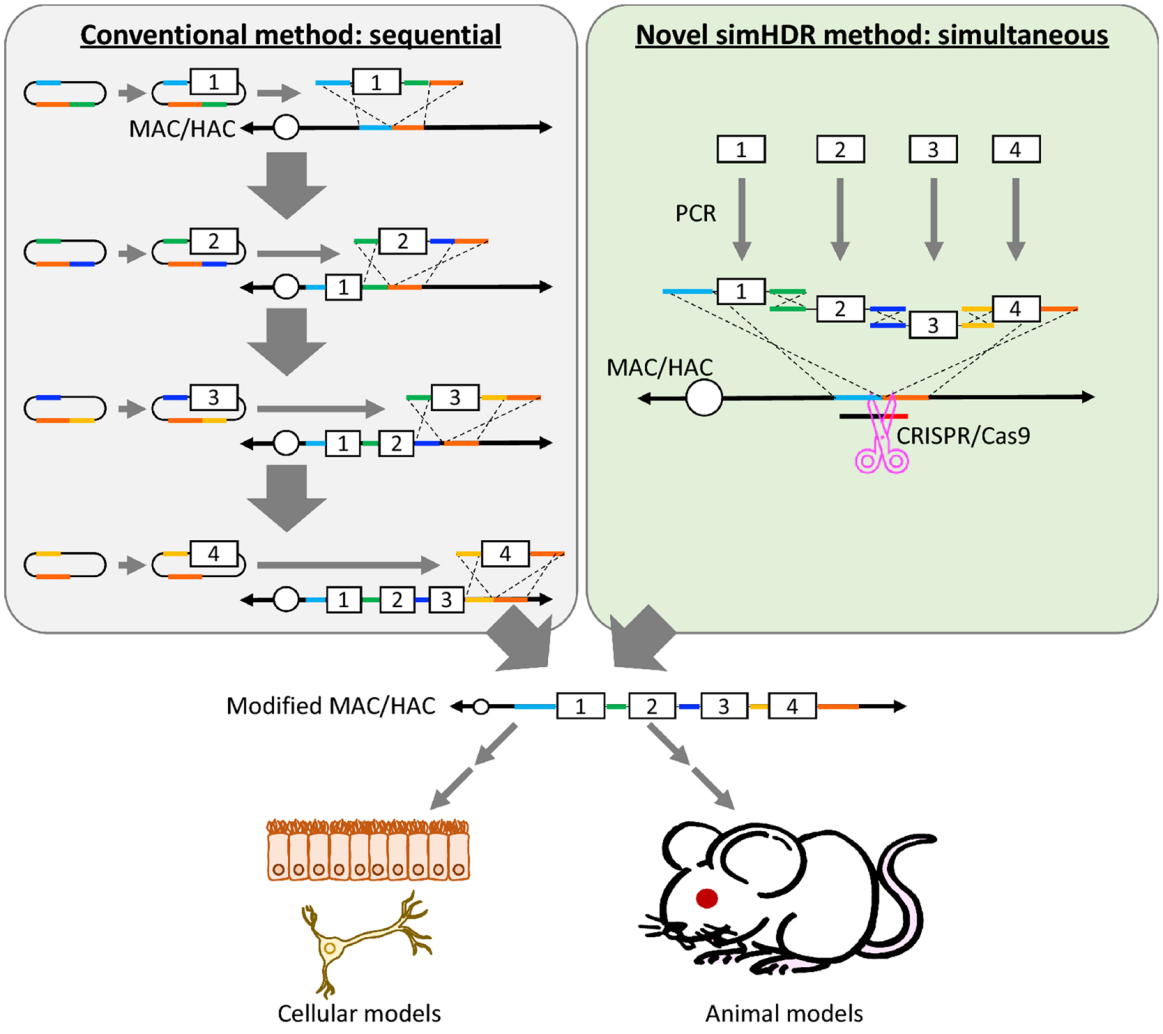
Schematic depiction of generating genetically modified models using MAC/HAC loaded with multiple HDR donors via conventional and novel methods. Conventional loading method of multiple HDR donors sequentially is shown in left. Novel loading method of multiple HDR donors simultaneously as reported in this study is shown in right. Constructed MAC/HAC contained multiple HDR donors that can subsequently be used for generating genetically modified cellular and animal models.

In Figure 4c, the text on the left side of the image “DT40-10MAC2-HLA-A” was incorrectly given as “CHO-10MAC2-HLA-A”. The original Figure 4 and accompanying legend appear below.

**Figure 4:**
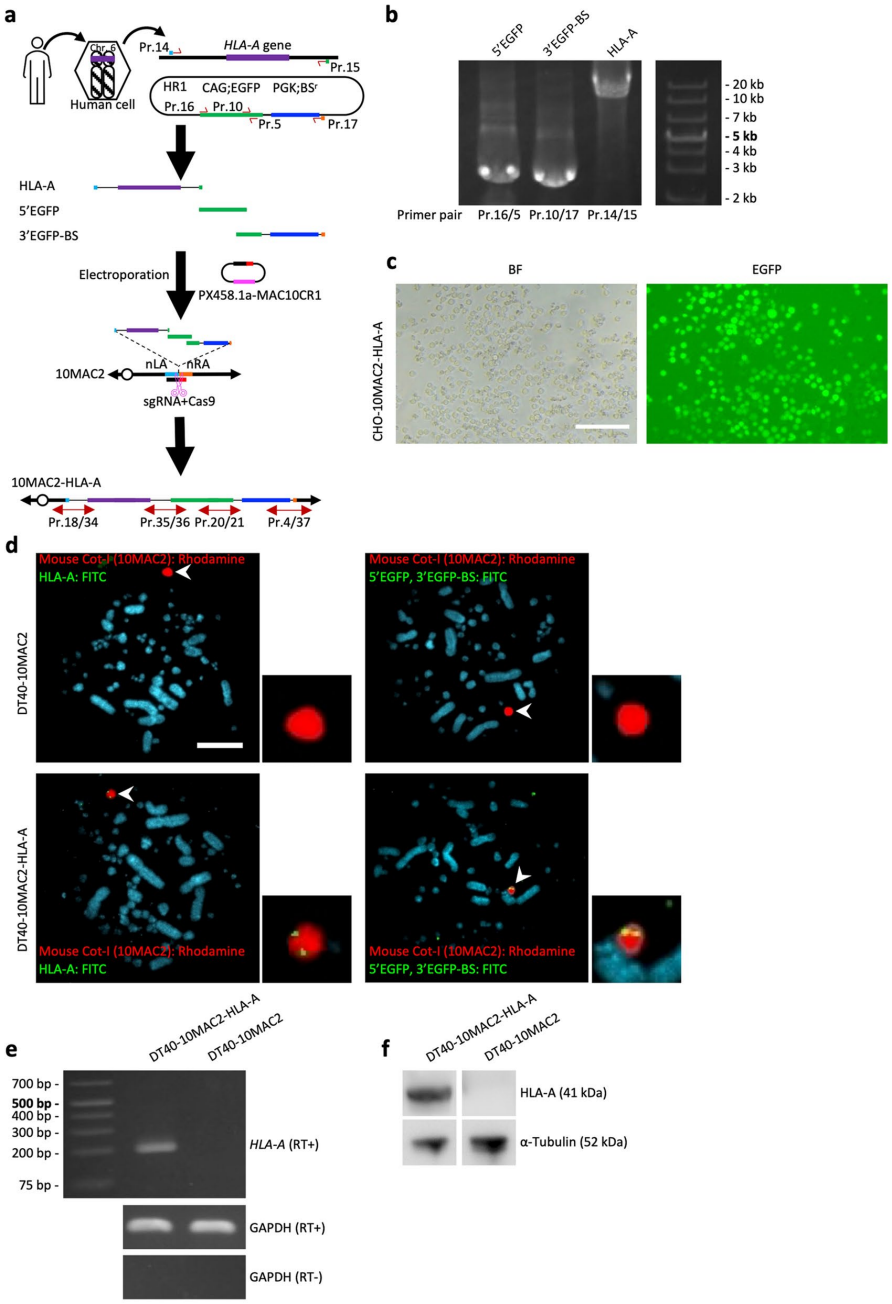
The simHDR based direct cloning of *HLA-A* genomic region. (**a**) Schematic representation of the simHDR illustrating the loading of genomic DNA sequence from human cells onto the 10MAC2. Arrows indicate the position of PCR primers used for analysis. (**b**) Preparation of PCR HDR donor fragments. Confirmation of precise amplification by electrophoresis. For gel source data, refer to Supplementary Fig. S15. (**c**) Image of DT40 cells carrying the 10NAC2-HLA-A. EGFP expression indicates the presence of the 10NAC2-HLA-A. BF, bright field. Scale bar: 100 µm. (**d**) Representative image of metaphase FISH analysis with mouse Cot-I (red) detecting 10MAC2 and HLA-A or 5’EGFP and 3’EGFP-BS PCR HDR donor fragments (green). Arrowhead indicates the 10MAC2 and the inset shows an enlarged image thereof. Scale bar: 10 μm. (**e**) RT-PCR products generated from DT40-10MAC2-HLA-A and DT40-10MAC2 cells cDNAs. Each end point PCR products are shown. For gel source data, refer to Supplementary Fig. S16. (**f**) Detecting HLA-A protein by western blotting. For membrane source data, refer to Supplementary Fig. S17.

In Supplementary Figure S4, the 289th (279th in the original file) base “T” indicated by the black arrowhead in the “HR1 plasmid_nLA” sequence was incorrectly given as “C”.

The original Article and accompanying Supplementary Information file have been corrected.

