# Supplementary material for: Simultaneous loading of PCR-based multiple fragments on mouse artificial chromosome vectors in DT40 cell for gene delivery
Source: Sci Rep. 2022 Dec 16;12:21790. doi: 10.1038/s41598-022-25959-9 (PMC9758134; doi:10.1038/s41598-022-25959-9)
Supplement: Supplementary file 1 — Supplementary Information. [file 41598_2022_25959_MOESM1_ESM.pdf]

Supplementary Information

Simultaneous loading of PCR-based multiple fragments on mouse artificial chromosome vectors in DT40 cell for gene delivery

Kyotaro Yamazaki, Kyosuke Matsuo, Akane Okada, Narumi Uno, Teruhiko Suzuki, Satoshi Abe, Shusei Hamamichi, Nanami Kishima, Shota Togai, Kazuma Tomizuka, Yasuhiro Kazuki

Yamazaki *et al.* Supplementary Figure S1

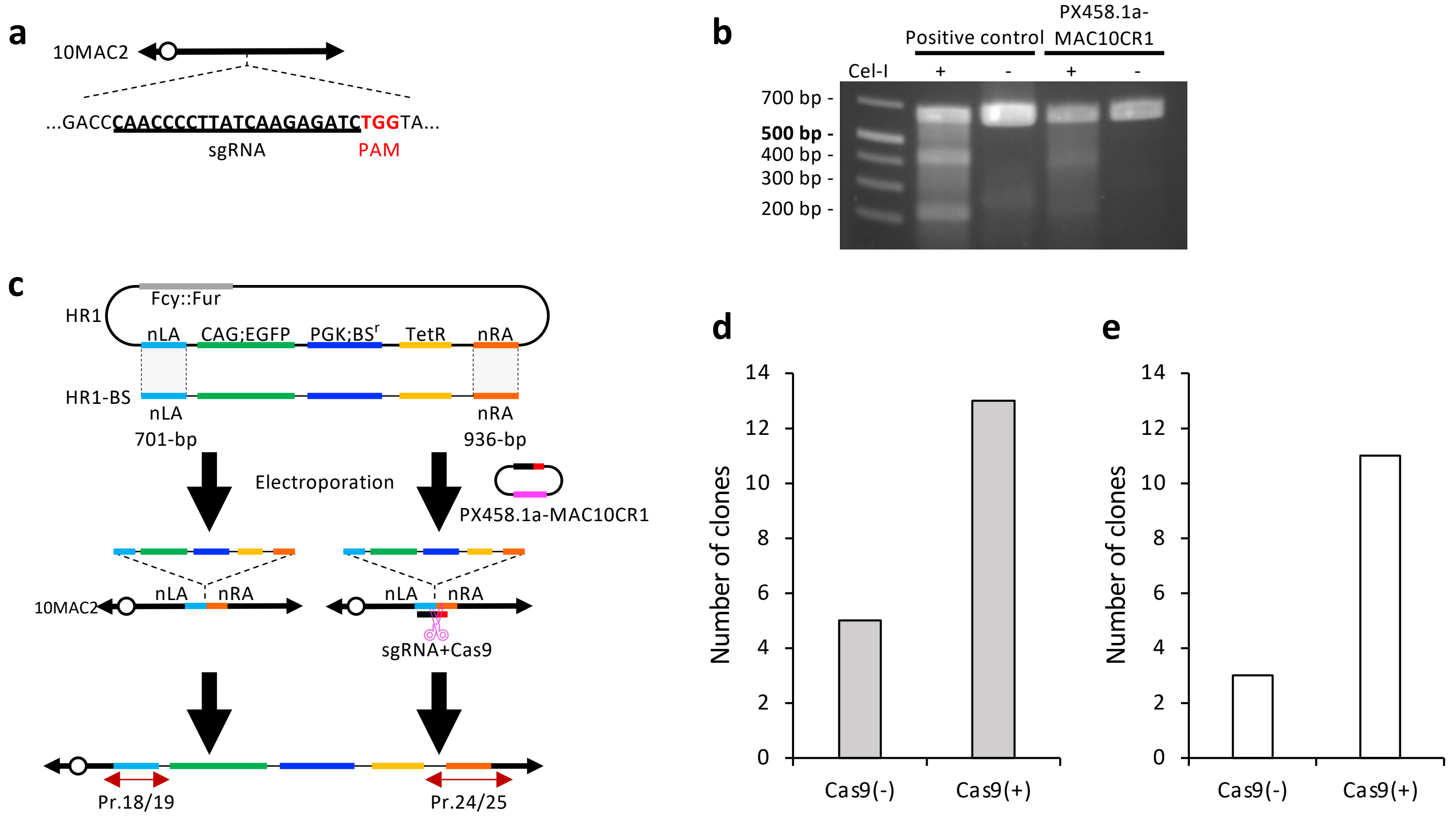

Supplementary Figure S1. Evaluation of sgRNA efficiency and CRISPR-Cas9 mediated KI efficiency in DT40 cells.

a, Diagrammatic representation of the 10MAC2 sequence and designed sgRNA recognition sequence. **b**, Analysis of sgRNA efficiency. Positive control was control heteroduplex/homoduplex product amplified using control G and C plasmid DNA contained in Survayor Mutation Detection Kit. For gel source data, refer to Supplementary Fig. S12. **c**, Schematic representation of HDR-KI with and without CRISPR-Cas9 mediated DSB. Arrows indicate the position of PCR primers used for analysis. Dashed lines and gray shadings indicate homology arm position and length. EGFP expression of DT40-10MAC2 cells containing recombined PCR-based HDR donors. **d**, The BS-resistant and EGFP-positive clone number after BS selection. **e**, Confirmation of precise simHDR by PCR analysis and the positive clone number.

Condition No.	DNA quantity (μg)	Voltage (V)	EGFP positive cells
1	0	275	0%
2	5	275	21%
3	10	275	29%
4	15	275	19%
5	20	275	11%
6	10	175	20%
7	10	225	38%
8	10	200	8%

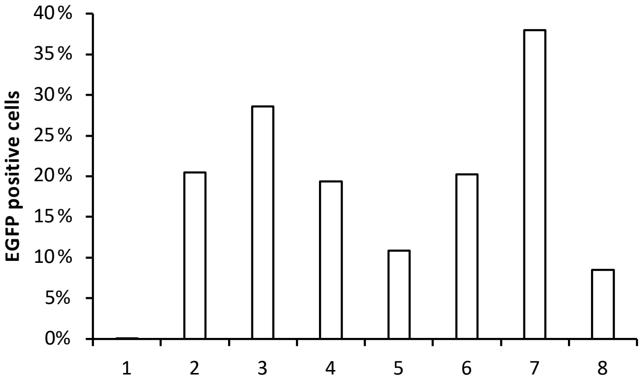

Supplementary Figure S2. Optimization of electroporation conditions.
To optimize the electroporation conditions for DT40-10MAC2 cells, pCX-EGFP was transfected and EGFP-positive rate was evaluated. Under all conditions, the number of cells (1 x 10^6 cells), buffer (Opti-MEM), and reaction volume (100 μL) are the same. The DNA quantities were compared for 5, 10, 15, and 20 μg. The voltages at poring pulse were compared for 175, 200, 225, and 275 V.

Yamazaki *et al.* Supplementary Figure S3

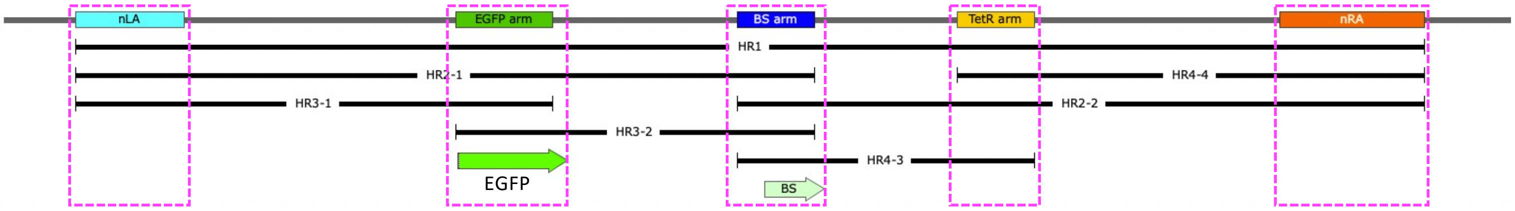

Supplementary Figure S3. Sequence analysis of 10MAC2 with HR1-BS, HR2-BSs, HR3-BSs, and HR4-BSs PCR HDR donor fragments.
Schematic diagram of 10MAC2 with HR1-BS, HR2-BSs, HR3-BSs, and HR4-BSs. Pink dotted lines indicate the positions of sequence analysis.

Yamazaki *et al.* Supplementary Figure S4

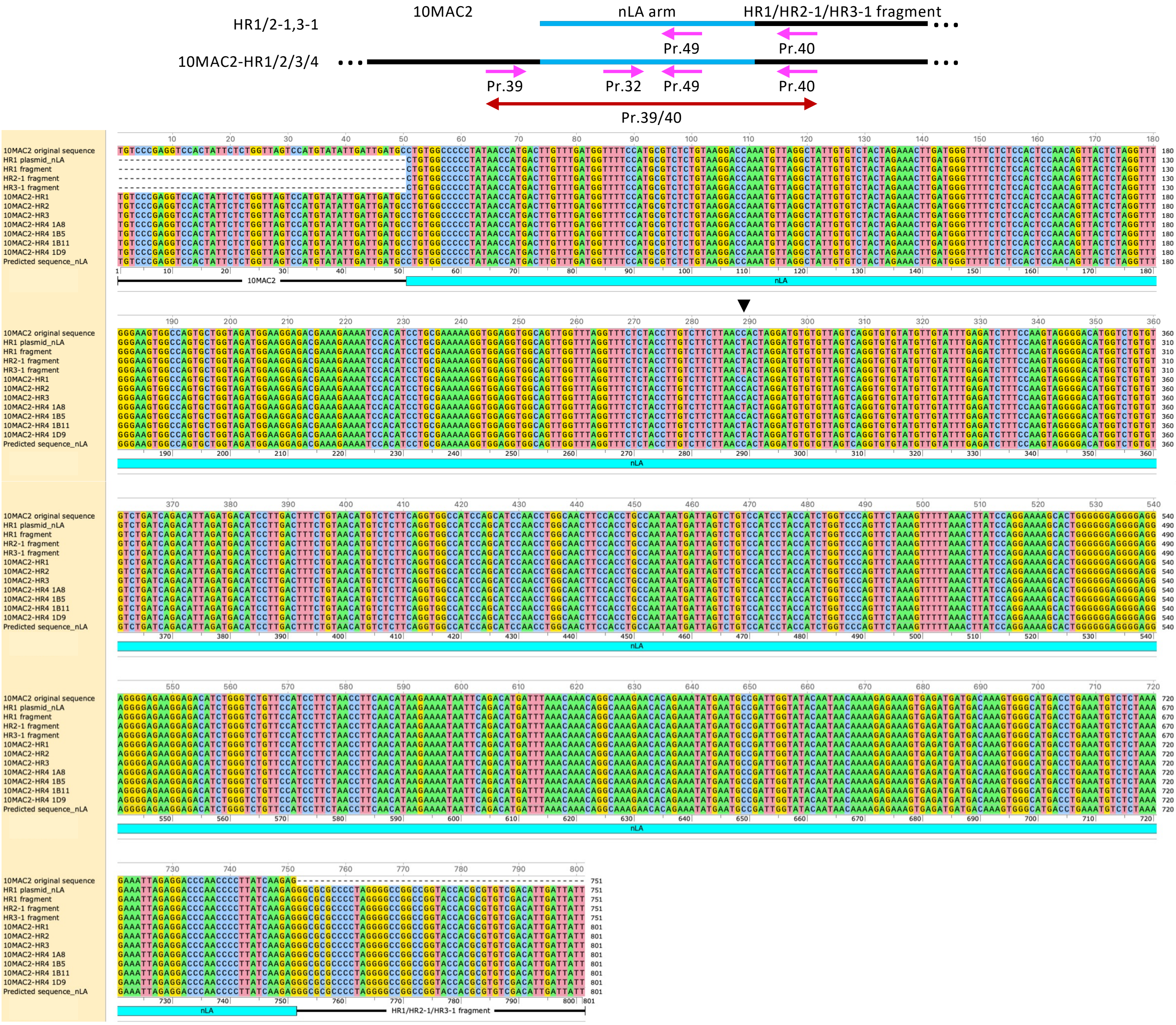

Supplementary Figure S4. Sequence analysis of 10MAC2 with HR1-BS, HR2-BSs, HR3-BSs, and HR4-BSs PCR HDR donor fragments.

Alignment of the nLA sequence of the PCR template HR1 plasmid vector and HR1/HR2-1/HR3-1 PCR HDR donor fragments, as well as the sequence of the 10MAC2-HR1/2/3/4 nLA junction. Black arrowheads indicate the positions of the single base substitution. Red arrows indicate PCR primers. Pink arrows indicate sequencing primers. The sequences were aligned by MUSCLE.

Yamazaki *et al.* Supplementary Figure S5

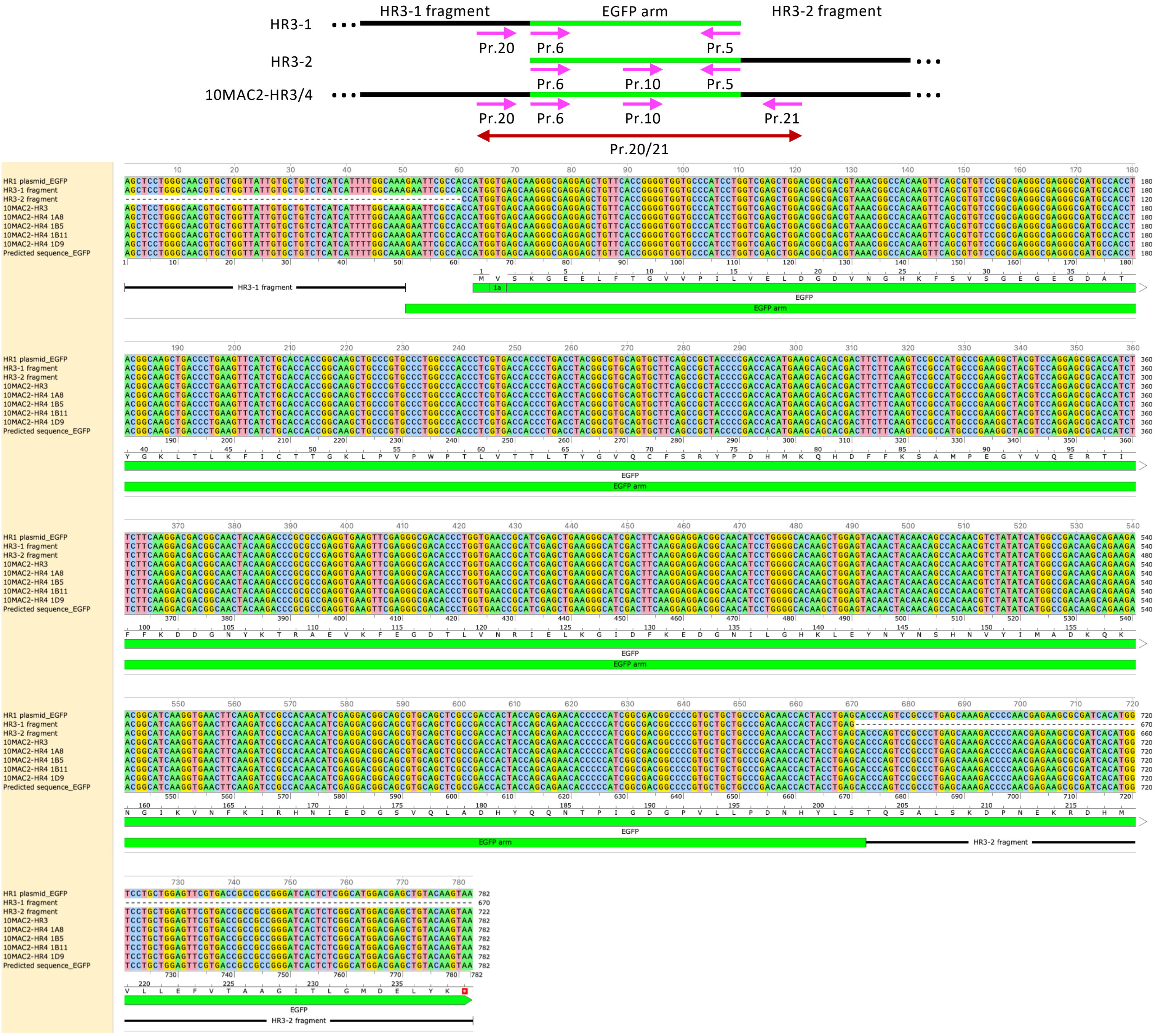

Supplementary Figure S5. Sequence analysis of 10MAC2 with HR3-BSs and HR4-BSs PCR HDR donor fragments.

Alignment of the EGFP arm sequence of the PCR template HR1 plasmid vector and HR3-1/3-2 PCR HDR donor fragments, as well as the sequence of the 10MAC2-HR3/4 EGFP junction. Red arrows indicate PCR primers. Pink arrows indicate sequencing primers. The sequences were aligned using MUSCLE.

Yamazaki *et al.* Supplementary Figure S6

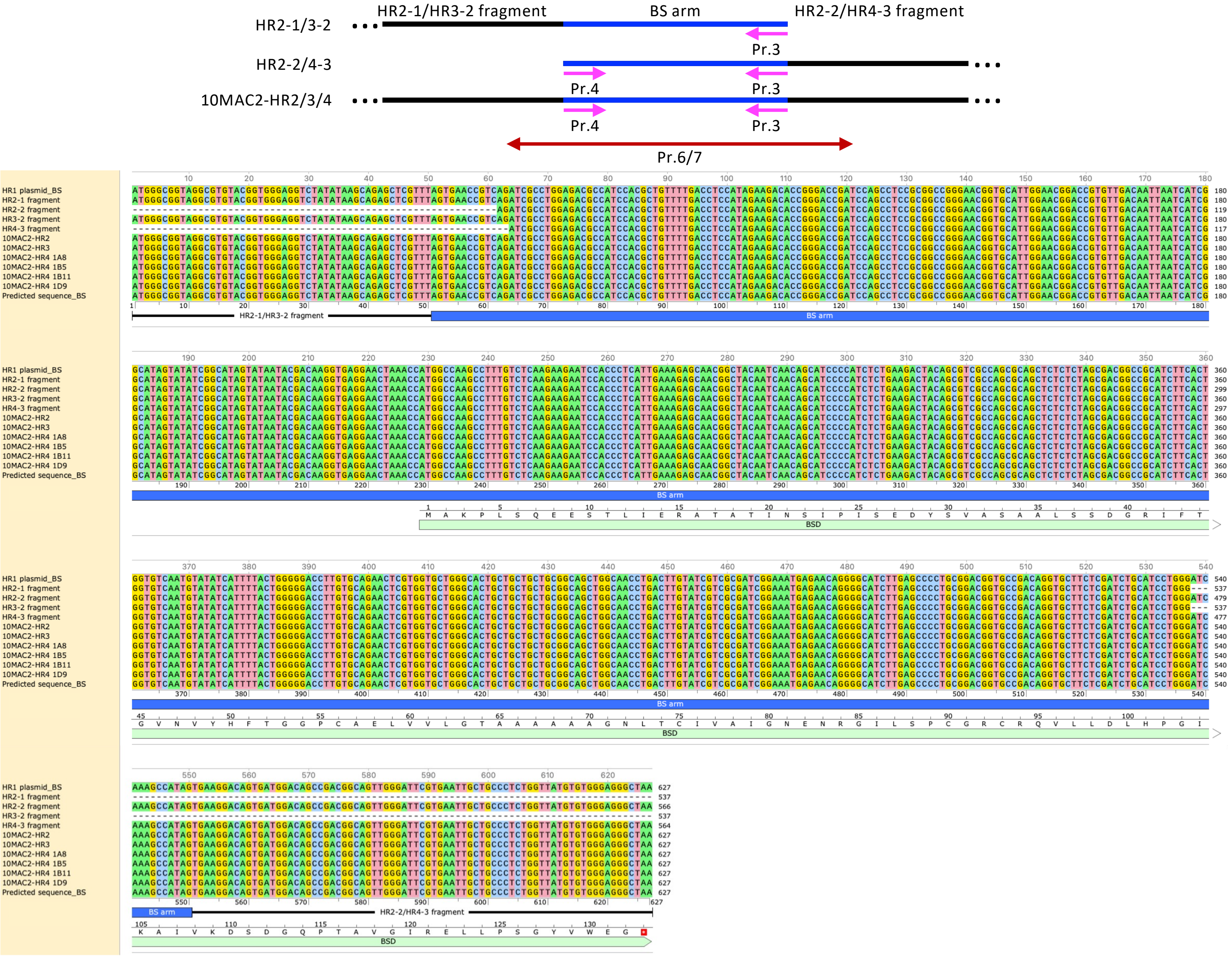

Supplementary Figure S6. Sequence analysis of 10MAC2 with HR2-BSs, HR3-BSs, and HR4-BSs PCR HDR donor fragments.
Alignment of the BS arm sequence of the PCR template HR1 plasmid vector and HR2-1/HR3-2/HR2-2/HR4-3 PCR HDR donor fragments, as well as the sequence of the 10MAC2-HR2/3/4 BS junction. Red arrows indicate PCR primers. Pink arrows indicate sequencing primers. The sequences were aligned using MUSCLE.

Yamazaki *et al.* Supplementary Figure S7

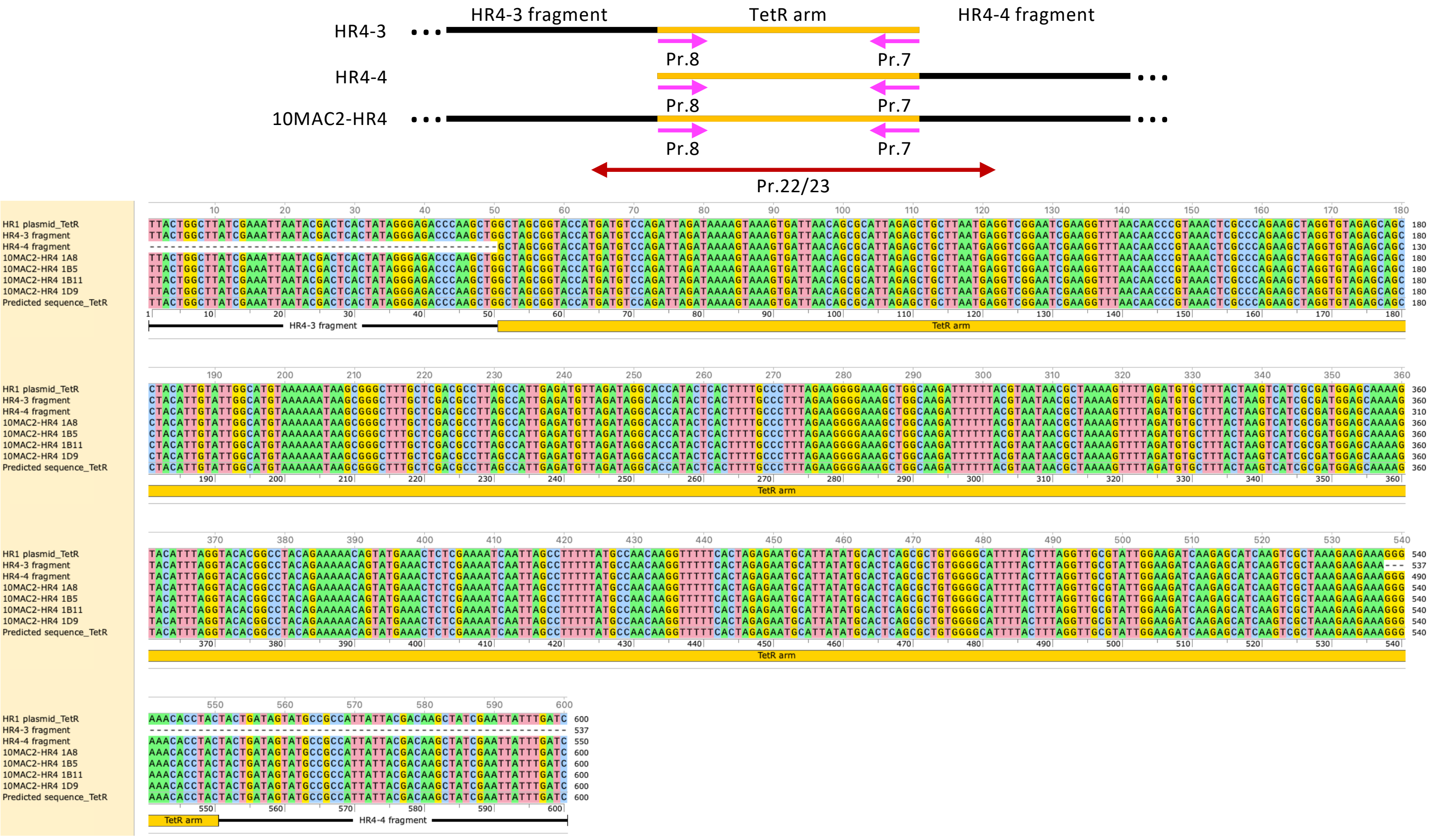

Supplementary Figure S7. Sequence analysis of 10MAC2 with HR4-BSs PCR HDR donor fragments.
Alignment of the TetR arm sequence of the PCR template HR1 plasmid vector and HR4-3/HR4-4 PCR HDR donor fragments, as well as the sequence of the 10MAC2-HR4 TetR junction. Red arrows indicate PCR primers. Pink arrows indicate sequencing primers. The sequences were aligned using MUSCLE.

Yamazaki *et al.* Supplementary Figure S8

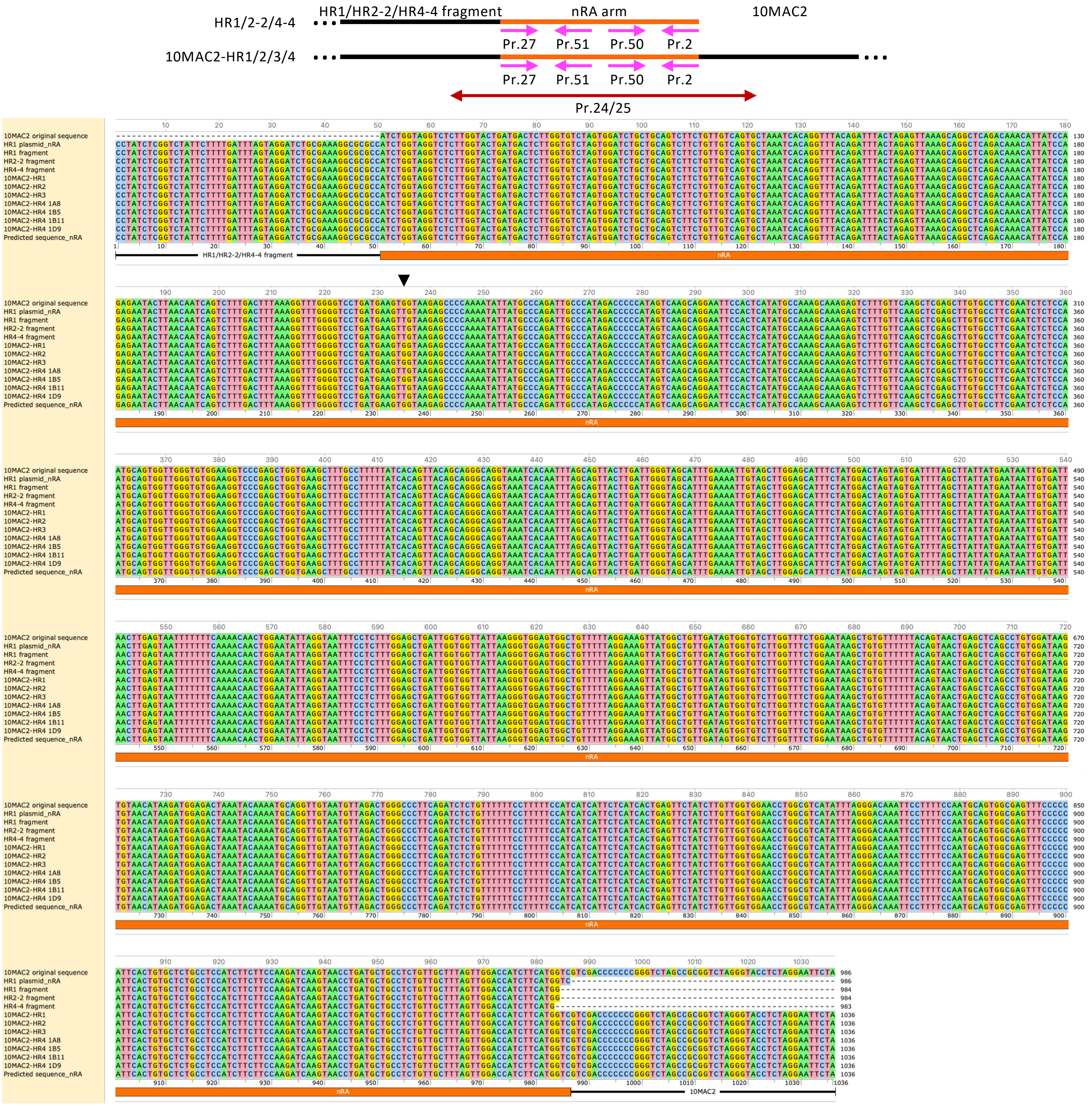

Supplementary Figure S8. Sequence analysis of 10MAC2 with HR1-BS, HR2-BSs, HR3-BSs, and HR4-BSs PCR HDR donor fragments.

Alignment of the nRA sequence of the PCR template HR1 plasmid vector and HR1/HR2-2/HR4-4 PCR HDR donor fragments, as well as the sequence of the 10MAC2-HR1/2/3/4 nRA junction. Black arrowheads indicate the positions of the single base substitution. Red arrows indicate PCR primers. Pink arrows indicate sequencing primers. The sequences were aligned using MUSCLE.

Yamazaki *et al.* Supplementary Figure S9

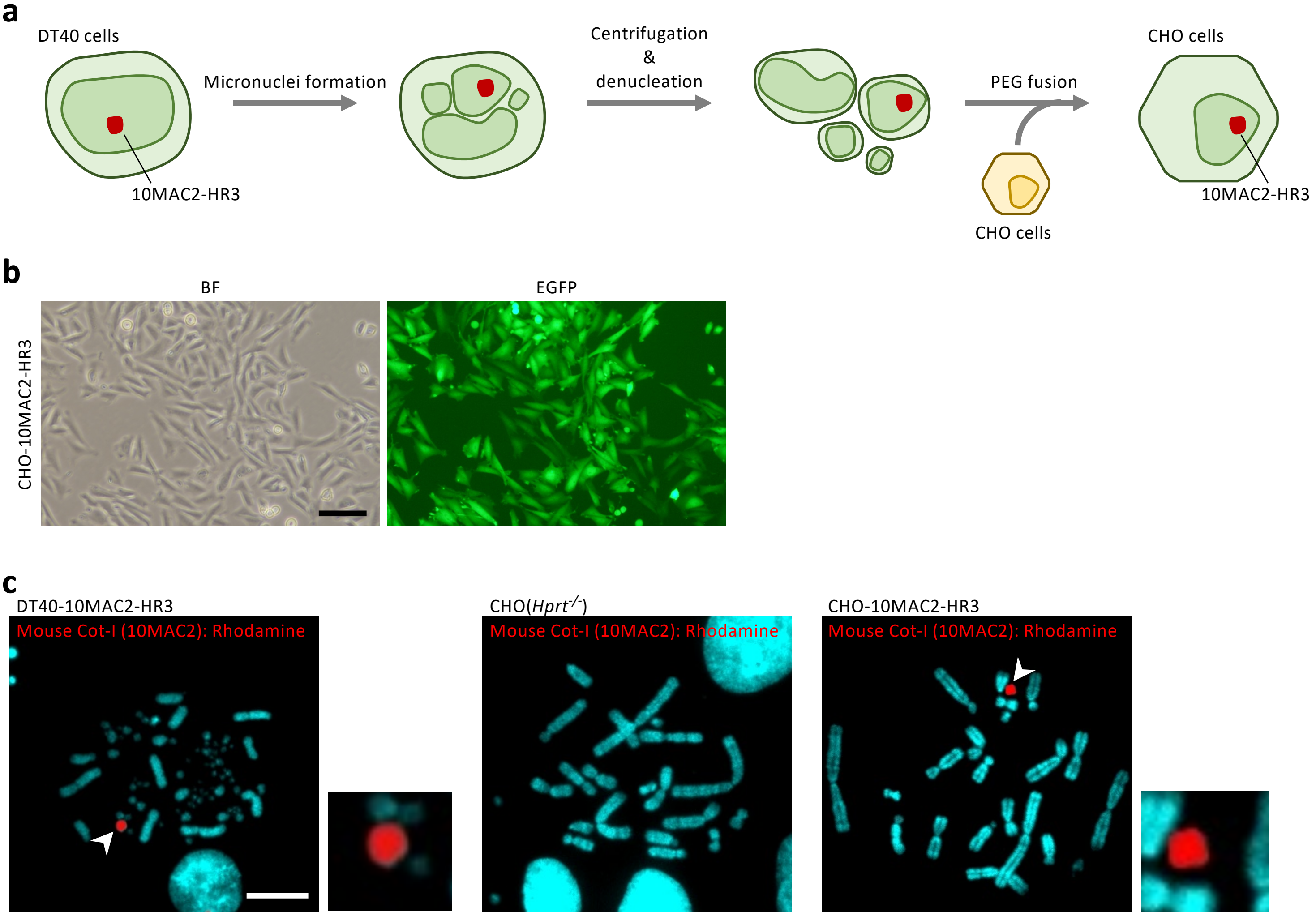

Supplementary Figure S9. Schematic depiction of MMCT and analysis of gene function loaded by the simHDR.

a, Schematic depiction of MMCT for transferring 10MAC2-HR3-BSs from DT40 to CHO cells. First, colcemid treatment induced micronuclei formation. Second, micronucleated cells were denucleated by centrifugation to obtain microcells. Finally, microcells were fused with recipient CHO cells by polyethylene glycol, and CHO clones expressing EGFP were obtained. **b**, Images of CHO cells carrying the 10NAC2-HR3-BSs. GFP expression indicates the presence of the 10NAC2-HR3-BSs. BF, bright field. Scale bar: 100 µm. **c**, Representative image of metaphase FISH analysis with mouse Cot-I (red) detecting the 10MAC2. Arrowhead indicates the 10MAC2 and the inset shows an enlarged image thereof. Scale bar: 10 µm.

Yamazaki *et al.* Supplementary Figure S10

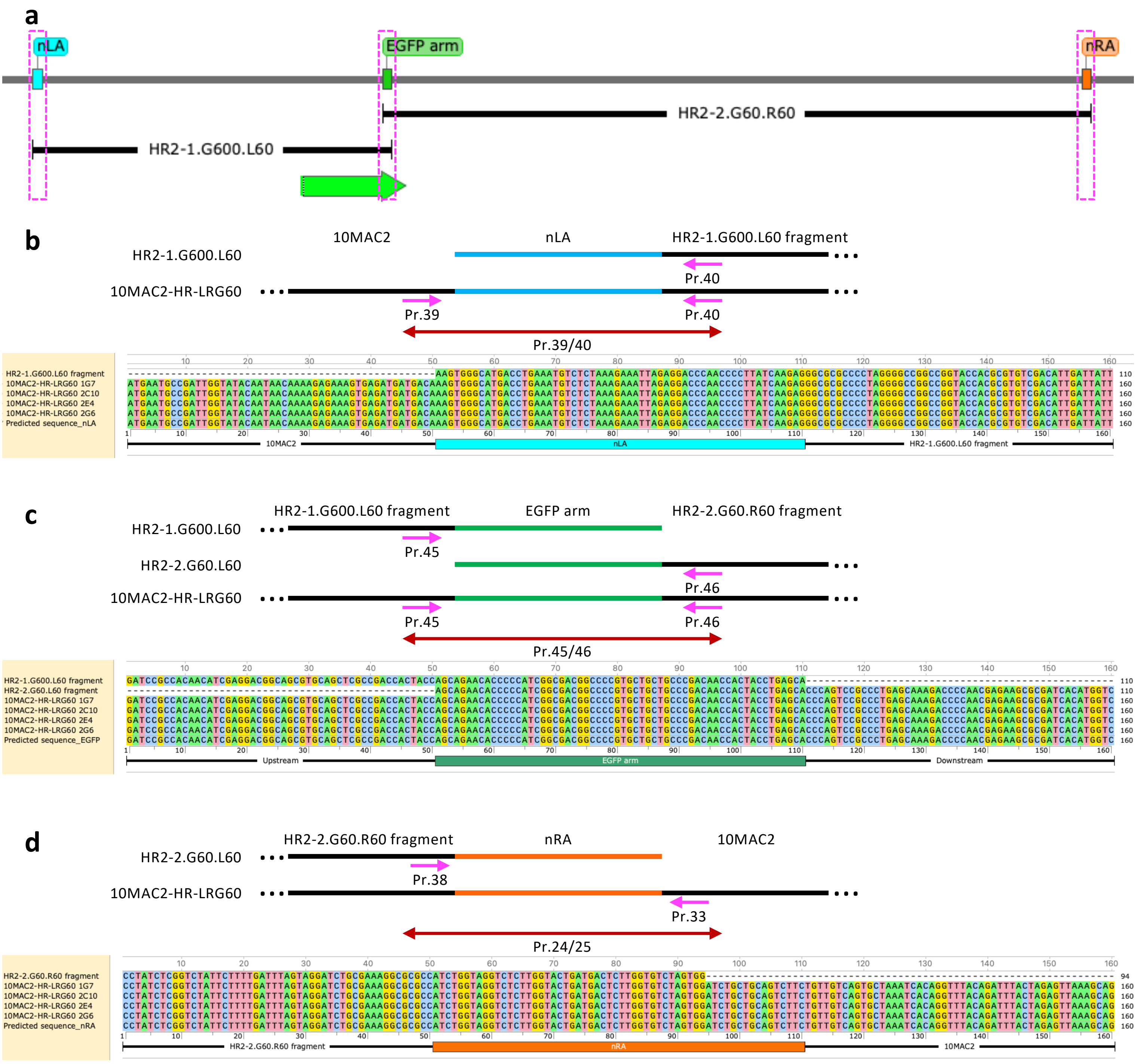

Supplementary Figure S10. Sequence analysis of 10MAC2 with HR-LRG60 PCR HDR donor fragments.

a, Schematic diagram of 10MAC2 with HR-LRG60. Pink dotted lines indicate the positions of sequence analysis. **b**, Alignment of the nLA sequence of the HR2-1.G600.L60 PCR HDR donor fragment, as well as the sequence of the 10MAC2-HR-LRG60 nLA junction. **c**, Alignment of the EGFP arm sequence of the HR2-1.G600.L60/HR2-2.G60.R60 PCR HDR donor fragments, as well as the sequence of the 10MAC2-HR-LRG60 EGFP junction. **d**, Alignment of the nRA sequence of the HR2-2.G60.R60 PCR HDR donor fragment, as well as the sequence of the 10MAC2-HR-LRG60 nRA junction. Red arrows indicate PCR primers. Pink arrows indicate sequencing primers. The sequences were aligned using MUSCLE.

Yamazaki *et al.* Supplementary Figure S11

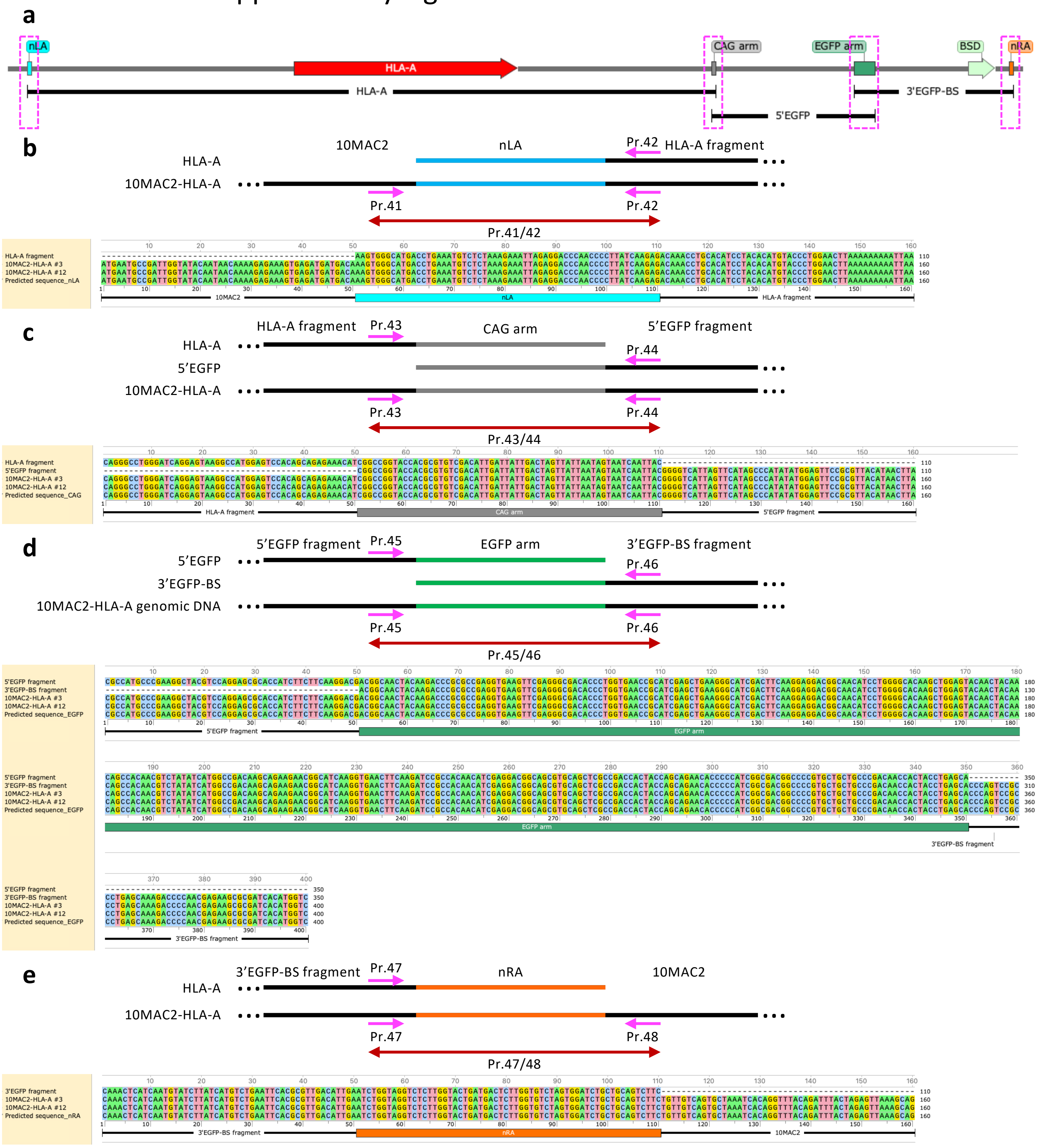

Supplementary Figure S11. Sequence analysis of 10MAC2 with HLA-A, 5'EGFP, and 3'EGFP-BS PCR HDR donor fragments.

a, Schematic diagram of 10MAC2 with HLA-A, EGFP, and BS. Pink dotted lines indicate the positions of sequence analysis. **b**, Alignment of the nLA sequence of the HLA-A PCR HDR donor fragment, as well as the sequence of the 10MAC2-HLA-A nLA junction. **c**, Alignment of the CAG arm sequence of the HLA-A/5'EGFP PCR HDR donor fragments, as well as the sequence of the 10MAC2-HLA-A CAG junction. **d**, Alignment of the EGFP arm sequence of the 3'EGFP-BS PCR HDR donor fragment, as well as the sequence of the 10MAC2-HLA-A EGFP junction. **e**, Alignment of the nRA sequence of the 3'EGFP-BS PCR HDR donor fragment, as well as the sequence of the 10MAC2-HLA-A nRA junction. Red arrows indicate PCR primers. Pink arrows indicate sequencing primers. The sequences were aligned using MUSCLE.

Yamazaki *et al.* Supplementary Figure S12

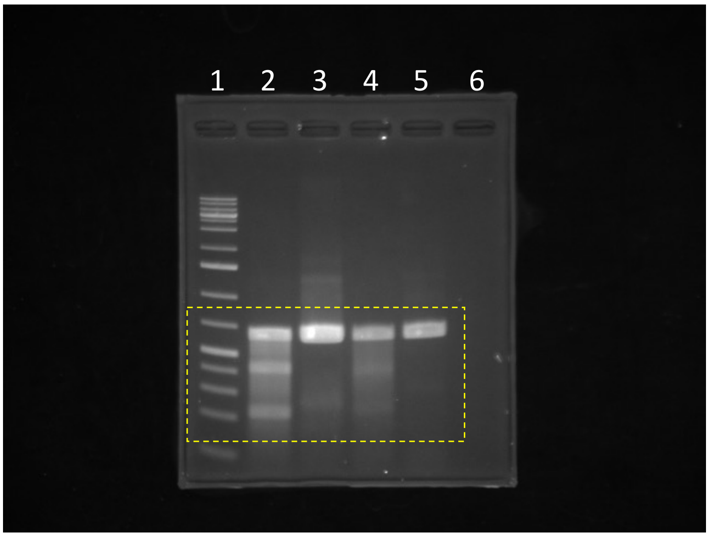

Lane		Cel-I	bp Homo	bp Hetero
1	Marker			
2	Kit positive control	+	633	415
3	Kit positive control	-		218
4	DT40-10MAC2	+	638	409
5	DT40-10MAC2	-		229
6				

Supplementary Figure S12. Uncropped images of electrophoresis.

Uncropped image of electrophoresis is presented, and the dotted square indicates the figure used in Supplementary Fig. S1b.

Yamazaki *et al.* Supplementary Figure S13

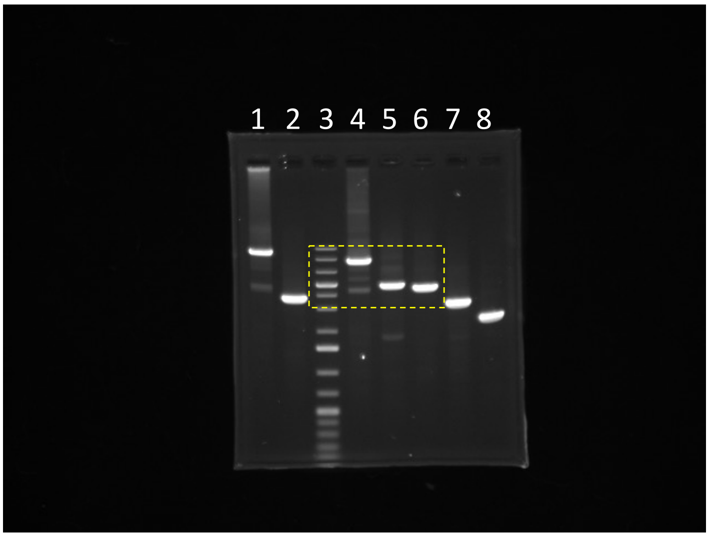

Lane		bp
1		
2		
3	Marker	
4	HR1	8711
5	HR2-1	4776
6	HR2-2	4435
7		
8		

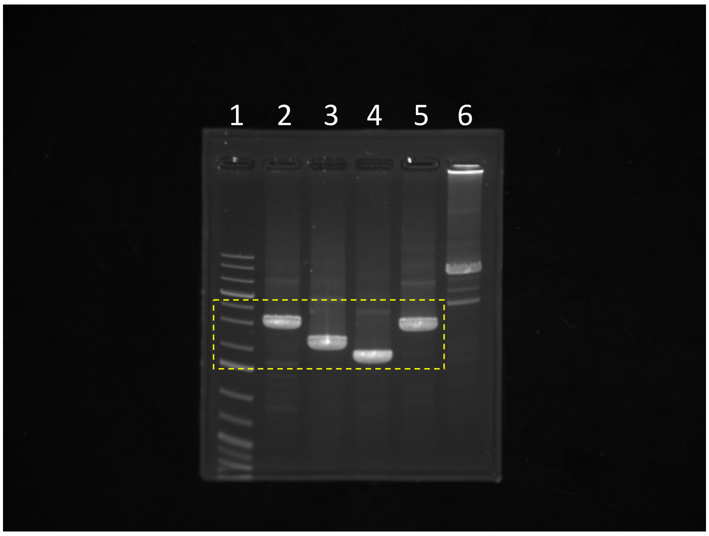

Lane		bp
1	Marker	
2	HR3-1	3085
3	HR3-2	2313
4	HR4-3	1921
5	HR4-4	3014
6		

Supplementary Figure S13. Uncropped images of electrophoresis.
Uncropped images of electrophoresis are presented, and the dotted squares indicate the figures used in Fig. 2b.

Yamazaki *et al.* Supplementary Figure S14

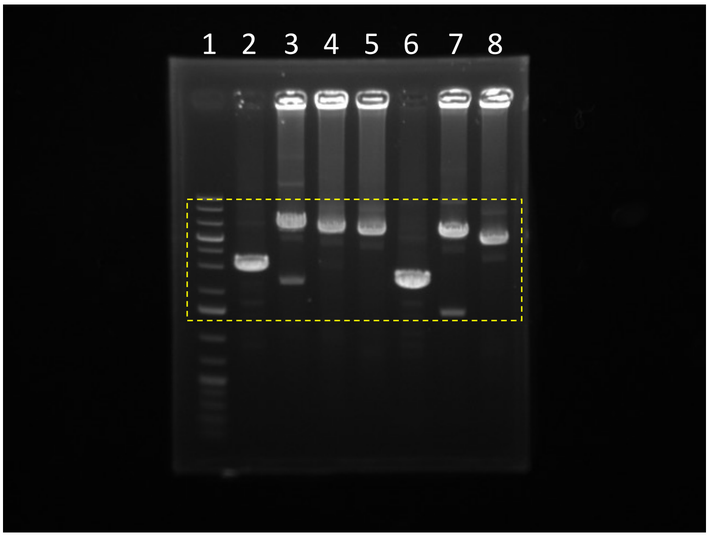

Lane		bp
1	Marker	
2	HR2-1.G600	3085
3	HR2-2.G600	6262
4	HR2-2.G300	5926
5	HR2-2.G60	5686
6	HR2-1.G600.L60	2436
7	HR2-2.G600.R60	5386
8	HR2-2.G60.R60	4810

Supplementary Figure S14. Uncropped images of electrophoresis.
Uncropped image of electrophoresis is presented, and the dotted square indicates the figure used in Fig. 3b.

Yamazaki *et al.* Supplementary Figure S15

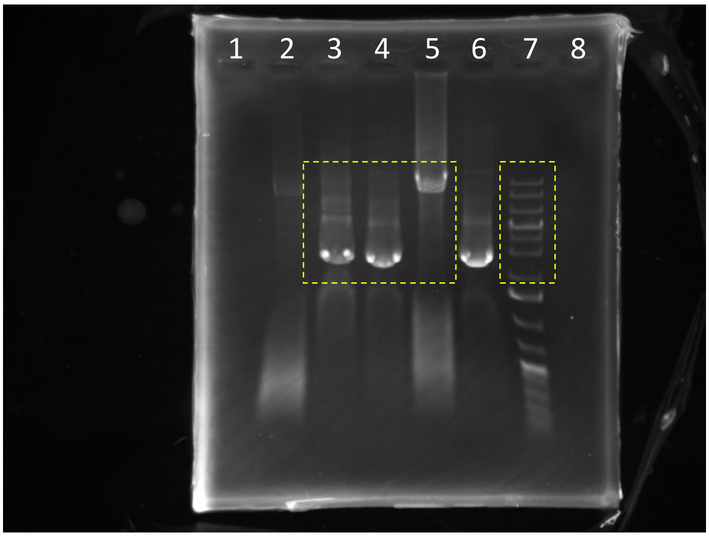

Lane		bp
1		
2		
3	5’EGFP	3085
4	3’EGFP-BS	2306
5	HLA-A	9977
6		
7	Marker	
8		

Supplementary Figure S15. Uncropped images of electrophoresis.
Uncropped image of electrophoresis is presented, and the dotted squares indicate the figures used in Fig. 4b.

Yamazaki *et al.* Supplementary Figure S16

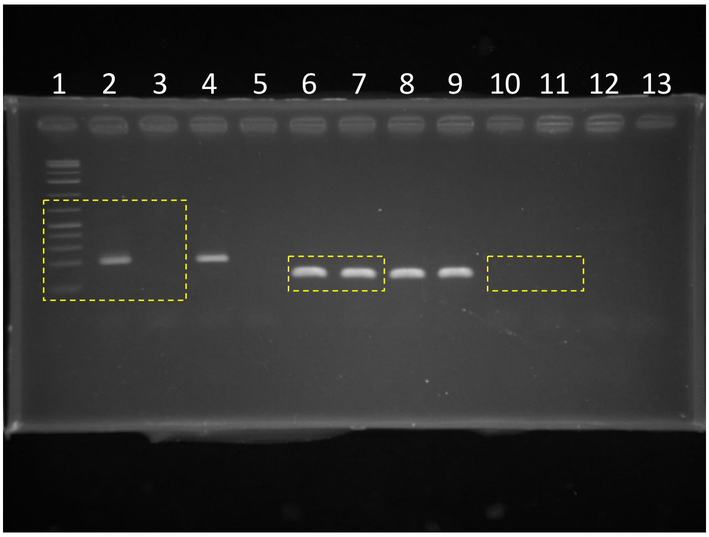

Lane		bp	Target	RT
1	Marker			
2	DT40-10MAC2-HLA-A	197	HLA-A	+
3	DT40-10MAC2	197	HLA-A	+
4				
5				
6	DT40-10MAC2-HLA-A	113	GAPDH	+
7	DT40-10MAC2	113	GAPDH	+
8				
9				
10	DT40-10MAC2-HLA-A	113	GAPDH	-
11	DT40-10MAC2	113	GAPDH	-
12				
13				

Supplementary Figure S16. Uncropped images of electrophoresis.
Uncropped image of electrophoresis is presented, and the dotted squares indicate the figures used in Fig. 4e.

Yamazaki *et al.* Supplementary Figure S17

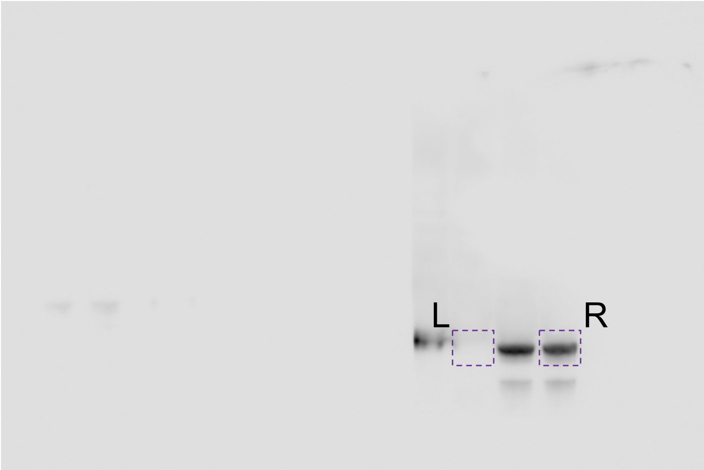

Sample position
L: DT40-10MAC2 total cell protein (20 μL)
R: DT40-10MAC2-HLA-A total cell protein (20 μL)

Condition
1st Ab: anti-HLA-A (Rabbit)
2nd Ab: anti-Rabbit (Goat)
Substrate: SuperSignal West Femto Maximum Sensitivity Substrate
Exposure: 30 s (Standard)

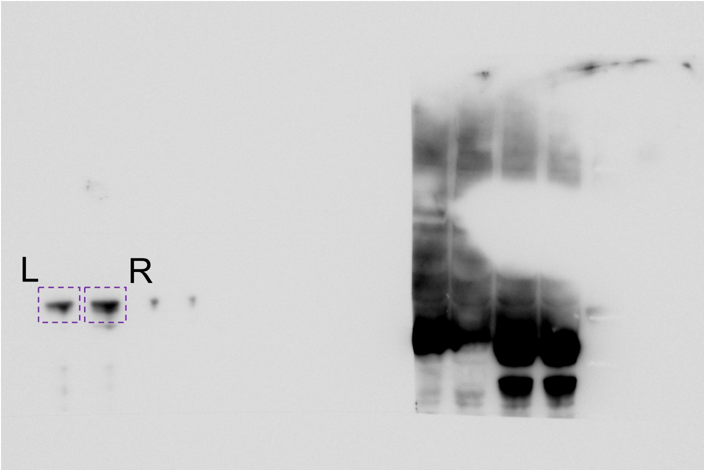

Sample position
L: DT40-10MAC2-HLA-A total cell protein (2 μL)
R: DT40-10MAC2 total cell protein (2 μL)

Condition
1st Ab: anti-α-Tubulin (Rabbit)
2nd Ab: anti-Rabbit (Goat)
Substrate: Pierce ECL Western Blotting Substrate
Exposure: 480 s (Standard)

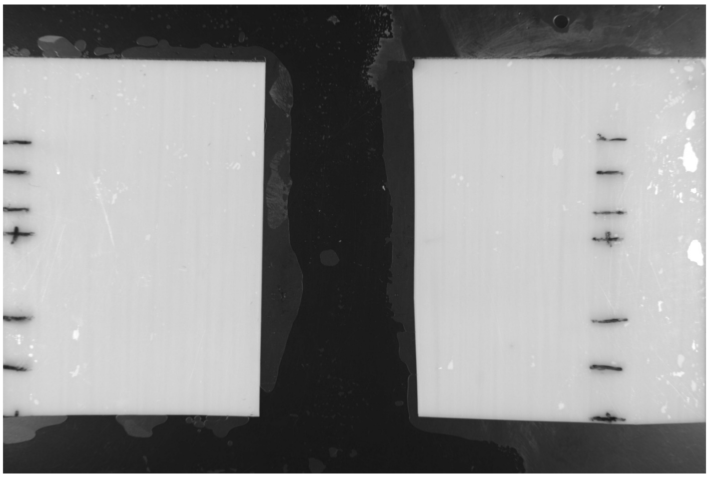

Supplementary Figure S17. Uncropped images of western blotting.
Uncropped images of western blotting are presented, and the dotted squares indicate the figures used in Fig. 4f.

Supplementary Table S1. Electroporation conditions related in Fig. 2 and Table 2.

	PX458	HR1	HR2-1	HR2-2	HR3-1	HR3-2	HR4-3	HR4-4	Total
HR1-BS	5,000	5,000							10,000
HR2-BSs	5,000		2,750	2,550					10,300
HR3-BSs	5,000			2,550	1,770	1,330			10,650
HR4-BSs	5,000				1,770	1,330	1,100	1,730	10,930

(ng)

	Poring pulse	Transfer pulse
Voltage (V)	225	20
Pulse length (ms)	1	50
Pulse interval (ms)	50	50
Number of pulses	2	5
Decay rate	10	40
Polarity	+	+/-

Supplementary Table S2. Electroporation conditions related in Fig. 3 and Table 3.

	PX458	HR2-1 .G600	HR2-2 .G600	HR2-2 .G300	HR2-2 .G60	HR2-1 .G600.L60	HR2-2 .G600.R60	HR2-2 .G60.R60	Total
HR-G600	5,000	1,770	3,600						10,370
HR-G300	5,000	1,770		3,400					10,170
HR-G60	5,000	1,770			3,260				10,030
HR-LR60	5,000					1,400	3,100		9,500
HR-LRG60	5,000					1,400		2,760	9,160

(ng)

	Poring pulse	Transfer pulse
Voltage (V)	225	20
Pulse length (ms)	1	50
Pulse interval (ms)	50	50
Number of pulses	2	5
Decay rate	10	40
Polarity	+	+/-

Supplementary Table S3. Electroporation conditions related in Fig. 4.

PX458	HLA-A	5'EGFP	3'EGFP-BS	Total
5,000	15,000	5,800	5,600	31,400

(ng)

	Poring pulse	Transfer pulse
Voltage (V)	225	20
Pulse length (ms)	1	50
Pulse interval (ms)	50	50
Number of pulses	2	5
Decay rate	10	40
Polarity	+	+/-

Electroporation were performed under conditions optimized for transfection efficiency of PCR HDR donors, 10-kb HLA-A, 3-kb 5’EGFP, and 2.3-kb 3’EGFP-BS.
